# Mitochondria: the central hub linking exercise to enhanced cardiac function

**DOI:** 10.3389/fphys.2026.1747133

**Published:** 2026-02-02

**Authors:** Jiaqin Cai, Tutu Wang, Shunchang Li

**Affiliations:** Sports Medicine Key Laboratory of Sichuan Province, Institute of Sports Medicine and Health, Chengdu Sport University, Chengdu, China

**Keywords:** exercise, heart, inflammatory response, mitochondria, oxidative stress

## Abstract

Sedentary lifestyle is a major risk factor for the occurrence and development of cardiovascular disease, which remains one of the leading contributors to global morbidity and mortality. Beyond inducing endothelial dysfunction, prolonged sedentary patterns trigger chronic inflammation and disrupt endogenous antioxidant defenses, resulting in mitochondrial dysfunction in cardiomyocytes and subsequent impairment of cardiac health. In contrast, regular physical exercise serves as an effective lifestyle intervention that mitigates sedentary-related cardiac damage and improves cardiac function. Mitochondria, as central organelles governing cellular survival and death, are thought to play a pivotal role in mediating the cardioprotective effects of exercise. However, the precise mitochondrial mechanisms underlying these benefits remain incompletely defined. This review aims to summarize current evidence on how exercise regulates mitochondrial function in the heart, with particular emphasis on recent advances linking mitochondrial respiration, dynamics, calcium homeostasis, inflammatory signaling, and oxidative stress to cardiac health. We further propose that exercise-induced improvements in mitochondrial function constitute a core mechanism underlying its cardioprotective effects. By comparing mitochondrial alterations under sedentary and exercise conditions, we provide a clearer mechanistic perspective on how lifestyle behaviors shape cardiac health. Furthermore, this paper also discusses signaling pathways that position mitochondria as key targets of exercise-induced cardiac protection.

## Graphical Abstract

**Figure:**
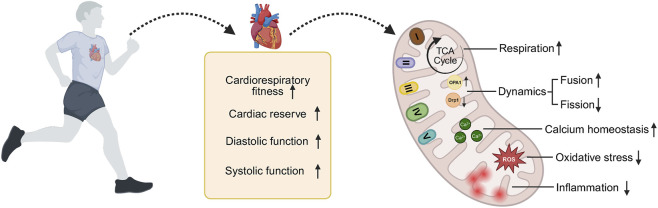

## Highlights

- Exercise improves cardiac energy metabolism efficiency by enhancing mitochondrial respiration function and dynamics in cardiomyocytes.
- Exercise exerts cardiac protection effects by improving calcium homeostasis in cardiomyocytes and enhancing antioxidant and anti-inflammatory capabilities.
- Exercise offers a non-pharmacological intervention strategy for sedentary-induced myocardial injury by targeting mitochondrial pathways.

## 1 Introduction

Regular exercise induces systemic adaptive responses that confer broad health benefits, making it an effective strategy for the prevention of various diseases (Dos Santos et al., 2022). However, physical inactivity due to sedentary lifestyles has become a widespread issue in modern society. It not only represents a major risk factor for cardiovascular disease but has also been listed by the World Health Organization (WHO) as one of the top ten leading causes of death in developed countries (Waseem et al., 2022). More importantly, the chronic disease burden resulting from inactivity continues to drive rising healthcare costs, making physical inactivity a critical public health issue that urgently needs to be addressed globally (Nguyen et al., 2022). As a non-pharmacological intervention, exercise offers distinct advantages—including cost-effectiveness, safety, and efficiency—in preventing cardiac disorders and enhancing cardiac function (Laranjo et al., 2024). Regular exercise can promote favorable cardiac remodeling, improve cardiac metabolism and performance, and reduce multiple risk factors associated with chronic diseases, thereby exerting protective effects on the heart and contributing to disease prevention (Chen et al., 2022). Recent studies further suggest that alterations in mitochondrial function within cardiomyocytes may play a central role in the mechanisms underlying exercise-induced cardio protection (Li et al., 2025a).

The heart is composed of multiple cell types, including cardiomyocytes, which are responsible for contractile function, as well as fibroblasts, endothelial cells, immune cells, lymphatic cells, and others that contribute to structural support, signaling regulation, and immune defense. Together, these diverse cell populations maintain cardiac structural integrity and physiological homeostasis (Feng et al., 2023; Xi et al., 2021). Among them, non-myocyte cell groups—such as fibroblasts, endothelial cells, and immune cells—play pivotal roles in exercise-induced cardioprotection. They participate in post-injury repair by suppressing fibrosis and collagen deposition, promoting vascular remodeling, and modulating immune responses, thereby preserving the stability of the cardiac microenvironment (Feng et al., 2023; Xi et al., 2021). Although cardiomyocytes account for only approximately 25%–35% of total cell number in the adult mammalian heart, they occupy nearly 70%–85% of the myocardial volume and are the primary executors of cardiac contractile function (Banerjee et al., 2007). Therefore, this review focuses mainly on mitochondrial function and its core mechanisms within cardiomyocytes, while also addressing the essential contributions of other cardiac cell types to exercise-mediated cardioprotection to provide a more comprehensive and integrated understanding.

Mitochondria are highly dynamic, double-membrane–bound organelles found in eukaryotic cells, serving not only as the primary site of adenosine triphosphate (ATP) synthesis but also as key regulators of cardiomyocyte survival and death through dynamic remodeling of their structure and function (Wei et al., 2025). Extensive studies have demonstrated that mitochondria safeguard cardiac energy supply and cellular homeostasis through classical mechanisms, including maintaining respiratory chain function, regulating redox balance, and participating in calcium handling. Collectively, these processes form the biological foundation of exercise-induced cardioprotection (Liang et al., 2024; da Silva et al., 2023). Consequently, the integrity of mitochondrial function is essential for preserving normal cardiac performance, whereas its impairment invariably leads to myocardial injury (Lu et al., 2019).

In addition to its benefits in healthy individuals, exercise also plays an irreplaceable role in the rehabilitation of cardiovascular diseases. Numerous studies have shown that regular physical activity promotes mitochondrial biogenesis, enhances mitochondrial respiration, and regulates the balance between mitochondrial fusion and fission, thereby significantly improving functional recovery following cardiac injury and facilitating the overall remodeling of cardiac performance (Ma et al., 2025b; Perera et al., 2025; Dubois et al., 2024). Therefore, research on exercise-induced cardioprotection should not only focus on adaptive mechanisms under healthy conditions but also emphasize its therapeutic value during post-disease recovery.

This review is based on a systematic search and screening of the literature published over the past two decades across PubMed, Web of Science, and Scopus, using keywords such as ‘exercise,’ ‘cardiac protection,’ ‘mitochondria,’ and ‘cardiomyocyte.’ Inclusion criteria for the literature were as follows: 1. The study is relevant to exercise, cardiac protection, and mitochondrial function; 2. The study provides experimental data or clinical evidence supporting its conclusions; 3. The study is published in a peer-reviewed journal; 4. The study types include laboratory research, clinical studies, clinical trials, and seminal review articles. Exclusion criteria for the literature were as follows: 1. The study employs flawed methodology or insufficient data support; 2. The study is not directly related to the topics of this review; 3. The study is not peer-reviewed or the source is unclear. We prioritized the inclusion of high-quality experimental studies, clinical evidence, and seminal review articles, and we integrated these findings in a structured manner to address the key scientific questions underlying exercise-mediated regulation of cardiac mitochondrial function and its protective effects. Although the beneficial effects of exercise on cardiac mitochondria have been well established, the precise mechanisms through which exercise regulates mitochondrial function to augment cardiac performance remain incompletely defined (Bishop et al., 2025). This article systematically summarizes the unique structural and physiological features of myocardial mitochondria, the relationship between mitochondria and cardiac health, the mitochondrial regulatory mechanisms underlying exercise-induced cardioprotection, and the roles of non-cardiomyocyte cells in mediating the cardioprotective effects of exercise. This review aims to explore the key mechanisms by which exercise-induced modulation of mitochondrial function in cardiomyocytes contributes to cardio protection.

## 2 Unique structure and physiological functions of cardiac mitochondria

### 2.1 Unique structural features of cardiac mitochondria

Adult cardiomyocytes are rich in mitochondria, which occupy approximately 30% of the cell volume and appear filamentous under the microscope (Wei et al., 2025). Based on their subcellular localization, cardiac mitochondria are generally categorized into subsarcolemmal mitochondria (SSM), interfibrillar mitochondria (IFM), and perinuclear mitochondria (PNM) (Lu et al., 2019). The mitochondrial structure comprises four compartments, arranged from the inside out as the matrix, inner membrane, intermembrane space, and outer membrane (Yang et al., 2021). The inner membrane contains cristae, which play a key role in oxidative phosphorylation, while the outer membrane regulates mitochondrial dynamics, cellular metabolism, and cell death (Gupta and Becker, 2021; Yang et al., 2021). The intermembrane space is enriched with proteins that serve as a dynamic buffer between the cytoplasm and the matrix (Yao et al., 2022). The mitochondrial matrix is relatively viscous, which affects molecular diffusion and enzymatic reaction efficiency. Furthermore, the metabolic state of cardiomyocytes is closely related to the physical and chemical properties of the matrix (Bulthuis et al., 2023).

In the healthy heart, mitochondria exhibit a highly organized architecture and stable functional capacity. Mitochondria in normal cardiomyocytes are relatively uniform in size, predominantly elongated in shape, and contain sufficient copies of mtDNA to ensure adequate expression of respiratory chain proteins and proper oxidative phosphorylation (Adams et al., 2023; Otten et al., 2020). Studies have shown that mitochondrial membrane potential is maintained at a relatively high level under physiological conditions, supporting robust cellular energy metabolism (Zou et al., 2021). In addition, the inner mitochondrial membrane features densely packed and well-organized cristae, a structural characteristic essential for efficient oxidative phosphorylation and sustained ATP production (Adams et al., 2023).

In diseased states, the structure and function of cardiac mitochondria are broadly compromised, leading to disrupted energy metabolism and impaired cellular performance. Mitochondria in pathological cardiomyocytes often exhibit swelling, fragmentation, or the formation of abnormally enlarged organelles, accompanied by a reduction in mtDNA copy number and suppressed expression of respiratory chain proteins, collectively resulting in diminished oxidative phosphorylation efficiency (Otten et al., 2020; Chaanine et al., 2019). Disease conditions also precipitate a decline in mitochondrial membrane potential, which in turn promotes excessive Reactive Oxygen Species (ROS) generation and further destabilizes cellular bioenergetics (Zou et al., 2021). At the same time, mitochondrial cristae become reduced in number and disorganized, changes that markedly impair oxidative phosphorylation and ATP production (Chaanine et al., 2019).

By comparing the effects of high-intensity interval swimming training versus sedentary conditions on myocardial mitochondrial ultrastructure in Sprague–Dawley (SD) rats, we found that, relative to the sedentary group, the exercise group exhibited significantly higher mitochondrial morphological metrics (circularity) and size metrics (mean area and perimeter) (Pasmiño et al., 2024). Transmission electron microscopy further revealed a reduced mitochondrial abundance in the sedentary group, accompanied by prominent structural abnormalities, including disorganized or even disrupted cristae and poorly defined outer membrane boundaries; in contrast, the exercise group showed increased mitochondrial abundance, with cristae arranged in a parallel and orderly manner and a sharply delineated outer membrane outline (Pasmiño et al., 2024).

### 2.2 Physiological functions of cardiac mitochondria

#### 2.2.1 Mitochondrial respiration

Oxygen consumption in the healthy human heart at rest is markedly higher than in other organs, reflecting its substantial energy demand (Sun et al., 2024). Cardiac energy supply is primarily in the form of ATP (Lopaschuk et al., 2021). However, the heart lacks the capacity to store ATP, energy must be replenished continuously; otherwise, ATP would be depleted within 2–10 s, ultimately leading to impaired cardiac function (Lopaschuk et al., 2021). Thus, the body must generate large amounts of ATP continuously to sustain cardiac contractility and various metabolic processes.

Mitochondria, as the primary site of ATP production, generate approximately 30 kg of ATP per day, with about 90% of the ATP utilized by the heart derived from mitochondria (Wei et al., 2025). A small fraction of cardiac ATP comes from glycolysis, whereas the majority is produced via fatty acid–driven OXPHOS (Sun et al., 2024). Fatty acids, serving as the main mitochondrial substrate for ATP production, are conjugated with coenzyme A to form fatty acyl-CoA within mitochondria. Through β-oxidation, fatty acyl-CoA is converted into acetyl-CoA, reduced flavin adenine dinucleotide (FADH_2_), and reduced nicotinamide adenine dinucleotide (NADH). FADH_2_ and NADH then enter the electron transport chain (ETC) to drive ATP synthesis, while acetyl-CoA enters the tricarboxylic acid (TCA) cycle to generate additional FADH_2_ and NADH, which further fuel ATP production via the ETC (Liu et al., 2023a).

The ETC, composed of mitochondrial complexes I, II, III, IV, and V, represents the fundamental machinery of OXPHOS (Guan et al., 2022). Complex I, the entry point of the ETC, catalyzes the oxidation of NADH to NAD^+^ (Vercellino and Sazanov, 2022). Dysfunction of Complex I directly impairs this process by reducing the electron-accepting capacity of its flavin mononucleotide (FMN) cofactor (Protasoni and Zeviani, 2021). Complex II plays a critical role in the TCA cycle by catalyzing the oxidation of succinate to fumarate, thereby enhancing cycle efficiency (Goetzman et al., 2023). Because Complex III constitutes the core of the respiratory chain, its structural and functional integrity is essential for maintaining mitochondrial respiration (Protasoni and Zeviani, 2021). Complex IV biogenesis relies on assembly factors encoded by nuclear DNA as well as structural subunits encoded by mitochondrial DNA. Mutations in critical genes from either source can disrupt proper Complex IV assembly, leading to dysfunction and ultimately to mitochondrial disease (Ghezzi and Zeviani, 2018).

Importantly, these mitochondrial complexes interact to form supercomplexes, which accelerate electron transfer, enhance mitochondrial respiratory efficiency, and simultaneously reduce reactive oxygen species (ROS) production from the ETC, thereby supporting normal cardiac contractile function (Guan et al., 2022).

#### 2.2.2 Mitochondrial dynamics

The integrity of the mitochondrial network is dynamically regulated by mitochondrial dynamics (Chen et al., 2023). This process primarily encompasses the complementary remodeling events of mitochondrial fusion and fission, while mitochondrial biogenesis increases mitochondrial abundance and mitophagy removes damaged organelles (Chen et al., 2023). Collectively, these dynamic processes govern the morphology, number, and quality of cardiac mitochondria (Chen et al., 2023). Dysregulation of mitochondrial dynamics impairs cardiac function and increases susceptibility to cardiovascular diseases, including arrhythmia, coronary artery disease, and heart failure (Hinton et al., 2024).

Mitochondrial fusion refers to the merging of two individual mitochondria into a larger organelle under the mediation of fusion proteins, thereby allowing mixing of their contents (Gao and Hu, 2021). This process involves both outer and inner membrane fusion (Gao and Hu, 2021). Outer membrane fusion is mediated by mitofusin 1 (Mfn1) and mitofusin 2 (Mfn2), and requires both mitochondria to express these proteins, whereas inner membrane fusion is driven by optic atrophy protein 1 (Opa1), which is sufficient on only one of the fusing mitochondria (Gao and Hu, 2021). Opa1 function is dynamically regulated through alternative splicing and proteolytic processing by Oma1 and Yme1L proteases, generating both membrane-anchored long isoforms and soluble short isoforms that coordinate inner membrane fusion in response to cellular conditions (Chan, 2020). Importantly, Yme1L enhances Opa1 cleavage under conditions of elevated OXPHOS, thereby promoting inner membrane fusion (Tokuyama and Yanagi, 2023). Tokuyama reported that loss of fusion proteins leads to mitochondrial fragmentation and impaired cardiac function (Tokuyama and Yanagi, 2023). Thus, mitochondrial fusion is essential for maintaining mitochondrial function and contributes to the healthy development of the mammalian heart.

Mitochondrial fission refers to the division of a single mitochondrion into two independent organelles, thereby redistributing its contents (Adebayo et al., 2021). In mammals, this process is primarily mediated by dynamin-related protein 1 (Drp1), a cytosolic GTPase that is specifically recruited to the mitochondrial surface by interacting with outer membrane receptors such as fission mitochondrial 1 protein (Fis1) and mitochondrial fission factor (MFF) (Adebayo et al., 2021). Because Drp1 lacks a mitochondrial targeting sequence, its recruitment is thought to depend on accessory proteins (Jin et al., 2021). Although hFis1 was initially proposed as a candidate receptor—based on the essential role of its yeast homolog Fis1 in promoting fission complex assembly on the mitochondrial outer membrane (Mozdy et al., 2000)—immunocytochemical analyses revealed that RNAi-mediated knockdown of hFis1 does not markedly alter Drp1 mitochondrial localization, suggesting that hFis1 is not indispensable for Drp1 recruitment (Lee et al., 2004). By contrast, Gandre-Babbe et al. demonstrated that MFF depletion suppresses carbonyl cyanide m-chlorophenylhydrazone (CCCP)–induced mitochondrial fission, with a much stronger effect than Fis1 (Gandre-Babbe and van der Bliek, 2008).

In addition to receptor-mediated recruitment, post-translational modifications of Drp1 critically regulate fission activity. For example, calcineurin-dependent dephosphorylation of Drp1 promotes its translocation to mitochondria, thereby facilitating fission (Chen et al., 2023). Functionally, fusion enables exchange of mitochondrial contents, allowing organelles to complement one another under metabolic or environmental stress and maintain optimal activity. Conversely, fission is essential for mitochondrial proliferation and quality control, as it enables selective removal of damaged components. The dynamic balance between these two opposing processes is crucial for sustaining normal mitochondrial function, and dysregulation of either fusion or fission contributes to the pathogenesis of various cardiac diseases (Adebayo et al., 2021).

#### 2.2.3 Mitochondrial calcium homeostasis

Mitochondria serve as a central regulatory hub for intracellular Ca^2+^ oscillations, largely owing to their high-capacity Ca^2+^ buffering ability (Hasan et al., 2024). Mitochondrial Ca^2+^ is now recognized as a pivotal second messenger that governs cardiomyocyte survival and death, and is critically involved in regulating OXPHOS, ROS generation, and mitophagy (Wei et al., 2025). Specialized channels located on the mitochondrial membranes control Ca^2+^ influx and efflux, thereby maintaining Ca^2+^ levels within an optimal range. This homeostatic regulation prevents pathological events such as mitochondrial permeability transition pore (mPTP) opening and mitochondrial swelling, which would otherwise compromise mitochondrial integrity and impair cardiac function (Xu et al., 2020).

Cytosolic Ca^2+^ uptake into mitochondria is primarily mediated by the mitochondrial calcium uniporter (MCU), an inner membrane channel that transports Ca^2+^ into the mitochondrial matrix (Hasan et al., 2024). Under respiratory conditions, however, when cytosolic Ca^2+^ exceeds ∼10 μM, the rate of mitochondrial Ca^2+^ uptake is no longer determined by MCU activity itself but instead limited by the rate at which the respiratory chain generates the proton gradient (Huang and Wilson, 2023). MCU activity is further modulated by a range of inhibitors, including noncompetitive ruthenium compounds and divalent cations that competitively bind to the Ca^2+^ recognition site (Huang and Wilson, 2023). In addition, certain metal ions can bind to specific MCU sites, altering its conformation and reducing Ca^2+^ affinity (Huang and Wilson, 2023).

To maintain mitochondrial Ca^2+^ levels within the physiological range, most matrix Ca^2+^ is extruded back to the cytosol via the mitochondrial sodium–calcium exchanger (NCX), while a smaller fraction exits through the mPTP. Notably, NCX overexpression enhances Ca^2+^ efflux from the matrix (Zhang et al., 2022). Beyond Ca^2+^ extrusion, NCX directly regulates cytosolic and mitochondrial Na^+^ and Ca^2+^ concentrations as well as membrane potential, while its own activity is subject to feedback control by these same factors. Moreover, Na^+^, Ca^2+^, and membrane potential act through multiple pathways to influence ROS homeostasis (Takeuchi and Matsuoka, 2021).

By taking up Ca^2+^, mitochondria stimulate OXPHOS and thereby boost ATP production to support cardiac contractility. However, Ca^2+^ overload induces cardiomyocyte death (Zhang et al., 2022). Thus, precise regulation of mitochondrial Ca^2+^ homeostasis is indispensable for sustaining cardiac energy metabolism and maintaining normal contractile function.

#### 2.2.4 Mitochondrial oxidative stress

Oxidative stress refers to a state of redox imbalance in which the production of ROS overwhelms the endogenous antioxidant defense systems (Liu et al., 2023b). ROS are generally considered byproducts of aerobic metabolism, and elevated ROS levels are a major driving force in the development of cardiovascular diseases (Liu et al., 2023b). While excessive ROS can impair mitochondrial function and cause cardiomyocyte injury, physiological levels of ROS act as critical signaling molecules that regulate multiple cellular pathways (Atici et al., 2023). Thus, tight regulation of ROS generation is required to ensure that physiological signaling pathways are activated while pathological ones are suppressed (Peoples et al., 2019).

Mitochondria not only represent the primary source of ROS but also harbor antioxidant defense systems that mitigate ROS accumulation (Isei et al., 2021). These include manganese superoxide dismutase, the glutathione peroxidase/reductase system, and the peroxiredoxin/thioredoxin system (Peoples et al., 2019; Isei et al., 2021). Moreover, Schulz et al. reported that hearts lacking mitochondrial uncoupling protein 3 (UCP3), which is located in the inner membrane, produce significantly higher levels of ROS compared with wild-type hearts. This finding suggests that UCP3 functions as a regulator of cardiac oxidative stress, and its overexpression may confer cardioprotective effects (Schulz and Schlüter, 2023).

Taken together, cardiac mitochondria safeguard the heart against oxidative stress–induced injury by tightly balancing ROS production and scavenging.

#### 2.2.5 Mitochondrial inflammatory response

Chronic inflammation promotes excessive ROS production and disrupts mitochondrial networks, thereby damaging the structure and function of cardiac mitochondria. This leads to impaired respiration, Ca^2+^ dysregulation, apoptosis, and ultimately, compromised cardiac function (Hinton et al., 2024). Mitochondria are now recognized as central hubs of proinflammatory signaling, playing pivotal roles in innate immune responses by engaging antimicrobial, antiviral, anti-infective, and cell injury pathways (Arslan et al., 2011; Andrieux et al., 2021). Upon pathogenic invasion, mitochondria release damage-associated molecular patterns (DAMPs), which in turn activate innate immunity. This process not only serves as a crucial component of host defense but is also tightly linked to the pathogenesis of diverse inflammatory diseases (Andrieux et al., 2021).

Importantly, mitochondria provide a critical platform for the activation of the NOD-like receptor thermal protein domain associated protein 3 (NLRP3) inflammasome (Andrieux et al., 2021). Multiple mechanisms converge to facilitate this process, including the translocation of cardiolipin from the inner to the outer mitochondrial membrane, where it directly interacts with NLRP3, and the role of mitochondrial ROS as key secondary messengers that initiate activation signals (Zong et al., 2024). Once activated, the NLRP3 inflammasome catalyzes the maturation of interleukin-1β (IL-1β) and interleukin-18 (IL-18), thereby driving inflammatory responses and inducing pyroptosis (Toldo and Abbate, 2024). Thus, suppression of NLRP3 inflammasome activation is critical for limiting inflammation.

Recent studies have suggested that mitochondrial quality control may be harnessed for anti-inflammatory interventions. For instance, D’Amico et al. demonstrated that urolithin A (UA), a gut microbiota–derived natural metabolite, enhances mitophagy and suppresses proinflammatory cytokine production, thereby exerting anti-inflammatory effects (D'Amico et al., 2021a). These findings indicate that mitophagy may serve as an important mechanism of inflammation resolution. Collectively, mitochondria represent promising therapeutic targets for inflammation control. Strategies that reduce mitochondrial ROS generation, inhibit NLRP3 inflammasome activation, and enhance mitophagy may suppress inflammatory pathways and confer cardio protection.

#### 2.2.6 Effects of sedentary lifestyle on cardiac mitochondrial function

In recent years, growing attention to the health consequences of sedentary behavior has led to an expanding body of evidence indicating that prolonged sedentariness can adversely affect cardiac mitochondrial function.

From the perspective of mitochondrial structural homeostasis, sedentariness may impair mitochondrial function early on by disrupting mitochondrial dynamics and Ca^2+^ homeostasis. Compared with exercise, sedentary behavior has been reported to significantly reduce MFN1 expression, whereas MFN2, OPA1 and DRP1 levels remain largely unchanged. This pattern suggests a selective suppression of mitochondrial fusion with relatively preserved fission, thereby disturbing the balance between fusion and fission and compromising mitochondrial dynamic homeostasis (No et al., 2020). Sedentariness also perturbs mitochondrial Ca^2+^ handling. In a rat model, Popoiu and colleagues showed that chronic sedentary conditions markedly attenuated Ca^2+^ uptake by cardiac mitochondria and reduced the efficiency of Ca^2+^-dependent control of mitochondrial respiration. Together, these alterations disrupt mitochondrial Ca^2+^ homeostasis and may contribute to impaired cardiac function (Popoiu et al., 2023). Collectively, such changes are expected to limit mitochondrial renewal and functional recovery, while increasing myocardial vulnerability under metabolic stress.

Functionally, sedentary behavior is characterized by both a reduced capacity for mitochondrial renewal and impaired bioenergetic efficiency. Compared with rats subjected to 8 weeks of swim training, long-term sedentariness increases oxidative stress in cardiac mitochondria and blunts activation of the insulin-like growth factor 1 (IGF1) signaling pathway, with reduced expression of key downstream targets, including protein kinase B (PKB/Akt) and glycogen synthase kinase-3β (GSK-3β). These changes are accompanied by a diminished capacity for mitochondrial biogenesis in the myocardium (Godoy Coto et al., 2024). In parallel, sedentariness decreases the activities of respiratory chain complexes II and IV, thereby reducing respiratory efficiency and weakening myocardial energy metabolism. As respiratory capacity declines, electron leak and reactive oxygen species (ROS) production are likely to rise, further increasing mitochondrial stress and exacerbating dysfunction (Perera et al., 2025).

**FIGURE 1:**
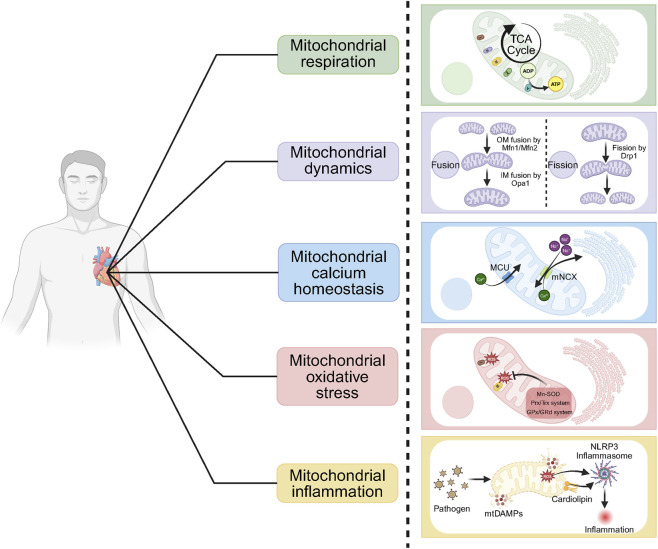
Physiological functions of cardiac mitochondria. The physiological roles of cardiac mitochondria include mitochondrial respiration, dynamics, calcium homeostasis, and resistance to oxidative stress and inflammatory responses. TCA, tricarboxylic acid cycle; I, NADH: ubiquinone oxidoreductase; II, succinate: ubiquinone oxidoreductase; III, ubiquinol: cytochrome c oxidoreductase; IV, cytochrome c oxidase; V, ATP synthase; ADP, adenosine diphosphate; ATP, adenosine triphosphate; Mfn1, mitofusin 1; Mfn2, mitofusin 2; Opa1, optic atrophy protein 1; Drp1, dynamin-related protein 1; MCU, mitochondrial calcium uniporter; NCX, sodium–calcium exchanger; ROS, reactive oxygen species; Mn-SOD, manganese superoxide dismutase; Prx/Trx, peroxiredoxin/thioredoxin; GPx/GRd, glutathione peroxidase/reductase; mtDAMPs, mitochondrial damage-associated molecular patterns; NLRP3 inflammasome, NOD-like receptor thermal protein domain–associated protein 3 inflammasome.

Ultimately, structural and functional mitochondrial perturbations tend to converge on oxidative stress and inflammatory signaling. Chronic sedentariness promotes excessive mitochondrial ROS generation in the myocardium, which can activate the NLRP3 inflammasome and stimulate the production and release of pro-inflammatory mediators such as IL-1β. This cascade supports the notion that sedentary behavior can trigger mitochondria-driven inflammatory responses by inducing mitochondrial dysfunction and amplifying oxidative stress, thereby exerting deleterious effects on cardiac function (Ding et al., 2024). Taken together, sedentariness compromises cardiac function through multiple, interconnected mechanisms, including reduced mitochondrial respiratory performance, disruption of mitochondrial dynamics and Ca^2+^ homeostasis, and heightened mitochondrial oxidative stress and inflammation, culminating in impaired overall cardiac function (Figure 1).

## 3 Mitochondria and cardiac health

A sedentary lifestyle has been identified as a major risk factor for cardiovascular disease, largely due to its detrimental effects on mitochondrial function in cardiomyocytes. Sedentary behavior reduces the body’s demand for oxidative metabolism, thereby suppressing mitochondrial biogenesis and leading to decreases in mitochondrial abundance, membrane potential, and oxidative phosphorylation capacity (Waseem et al., 2022). At the same time, sedentary behavior disrupts mitochondrial dynamics, resulting in fragmented mitochondrial networks, diminished respiratory chain activity, and increased ROS production, all of which impair energy supply and exacerbate cardiomyocyte injury (Tocantins et al., 2023). As these mitochondrial defects accumulate, both the structure and function of the myocardium become compromised, markedly elevating the risk of cardiovascular disease.

Regular physical activity plays a pivotal role in reversing the mitochondrial dysfunction in cardiomyocytes induced by prolonged sedentary behavior. Studies show that exercise increases myocardial oxygen demand, thereby enhancing mitochondrial oxidative phosphorylation and boosting ATP production to meet the energetic needs of the heart (Lopaschuk et al., 2010; Huynh and Pamenter, 2022). In addition, exercise modulates the expression of proteins governing mitochondrial fusion and fission, promoting fusion while limiting excessive fission, which collectively improves mitochondrial structure and function and confers cardioprotective effects (Haghighi et al., 2025). Consequently, regular exercise is essential for restoring cardiac energy metabolism, maintaining mitochondrial homeostasis, and reducing the risk of cardiovascular disease.

In summary, the effects of both sedentary and physically active lifestyles on cardiac health are largely mediated through mitochondrial function. As the central regulators of cardiac energy production, metabolic homeostasis, and cell survival, mitochondria are essential for maintaining normal myocardial performance. Consequently, mitochondrial functional status is closely tied to overall cardiac health.

## 4 Mitochondrial mechanisms of exercise-induced cardio protection

### 4.1 The role of mitochondrial respiration in exercise-induced cardio protection

The beneficial role of exercise in enhancing mitochondrial respiration and energy metabolism in the heart has been well documented. Lopaschuk et al. demonstrated that exercise increases myocardial oxygen consumption by 3- to 10-fold compared with the resting state in rats (Lopaschuk et al., 2010). This rise in oxygen consumption results from elevated adenosine diphosphate (ADP) concentrations, which drive mitochondrial OXPHOS and thereby accelerate ATP resynthesis (Huynh and Pamenter, 2022). Evidence has also shown that exercise promotes metabolic remodeling in the heart, particularly by enhancing mitochondrial oxidative metabolism to meet the increased energy demands during physical activity (Gibb and Hill, 2018). Burelle et al. provided additional evidence showing that treadmill-trained rats exhibit higher rates of glucose and palmitate oxidation compared with sedentary controls. This metabolic enhancement led to greater ATP production, which was associated with cardioprotective effects (Burelle et al., 2004). In another study, Suvorava et al. showed that exercise upregulates endothelial nitric oxide synthase (eNOS) in erythrocytes, the rate-limiting enzyme for nitric oxide (NO) synthesis. Increased NO production, in turn, enhanced mitochondrial respiration and contributed to cardioprotection (Suvorava and Cortese-Krott, 2018). Moreover, exercise training has been shown to upregulate cytochrome c oxidase subunit 4 (COX4) and nicotinamide phosphoribosyl transferase (NAMPT), while activating AMP-activated protein kinase (AMPK) and mitochondrial complex V. Together, these adaptations strengthen mitochondrial respiration, elevate myocardial energy levels, promote OXPHOS, and reduce the accumulation of harmful metabolic byproducts, thereby preserving cardiac function (Liang et al., 2024). In addition, a study on voluntary exercise training in high-fat diet-fed mice demonstrated that exercise significantly enhanced the activity of mitochondrial complex I, thereby improving the overall function of the electron transport chain and increasing oxidative phosphorylation efficiency. Furthermore, exercise increased the cardiac levels of cardiolipin, a critical lipid for maintaining efficient mitochondrial respiration, which further enhanced mitochondrial respiration capacity and ultimately improved cardiac function in obese mice (Perera et al., 2025). A growing body of research indicates that different types of exercise can induce specific and diverse adaptations in mitochondria. Specifically, training volume appears to be a key factor influencing mitochondrial content, while exercise intensity is a critical determinant of changes in mitochondrial respiration (Granata et al., 2018). One study examined the effects of repeated low-intensity (20 Hz) and high-intensity (100 Hz) stimulation on mitochondrial content and function. The study found that while mitochondrial content, as measured by citrate synthase activity, increased under both 20 Hz and 100 Hz stimulation, only the 100 Hz stimulation led to an increase in mitochondrial respiratory function, the content of respiratory chain complexes, and the assembly of mitochondrial supercomplexes (Yamada et al., 2021). Another human trial demonstrated that a 4-week sprint interval training (SIT) program increased mitochondrial respiration in muscle fibers by 25%, whereas no changes were observed with aerobic training (Granata et al., 2016). These results suggest that exercise can induce mitochondrial respiratory adaptations, with exercise intensity potentially being one of the key factors driving these changes. Additionally, Li et al. found that exhaustive exercise leads to a reduction in the activity of mitochondrial complexes I, II, and IV, thereby impairing the normal function of the electron transport chain and reducing oxidative phosphorylation efficiency. This process significantly weakens myocardial mitochondrial respiratory function, ultimately affecting cardiac energy metabolism (Li et al., 2022).

Collectively, these findings indicate that exercise training improves myocardial oxygen consumption, promotes metabolic remodeling, and upregulates energy metabolism–related enzymes, ultimately enhancing mitochondrial respiratory function and conferring cardioprotection.

### 4.2 The role of mitochondrial dynamics in exercise-induced cardio protection

Exercise has long been recognized as an effective strategy to prevent physical inactivity–induced cardiovascular disease, and accumulating evidence supports its regulatory effects on cardiac mitochondrial dynamics (Viloria et al., 2022; Haghighi et al., 2025). Studies have shown that exercise promotes mitochondrial fusion while inhibiting excessive fission, thereby maintaining elongated mitochondrial networks (Campos et al., 2023). This structural adaptation prevents mitochondrial fragmentation–induced oxidative stress, ATP synthesis impairment, and apoptosis, ultimately enhancing the metabolic capacity and adaptability of cardiac mitochondria under physiological stress conditions (Campos et al., 2023; Disatnik et al., 2013; Duan et al., 2021).

In an 8-week animal study, Haghighi et al. demonstrated that both aerobic and resistance training regulate the gene expression of key markers of mitochondrial fission and fusion. Specifically, the expression of Mfn1, Mfn2, and Opa1 was significantly increased, while Drp1 expression was reduced, indicating that exercise favors fusion over fission to ameliorate sedentary-induced mitochondrial dynamics imbalance (Haghighi et al., 2025; Hernandez-Resendiz et al., 2023). An additional 8-week animal study demonstrated that swimming training ameliorates mitochondrial dynamic abnormalities in rats with pressure overload–induced left ventricular dysfunction. Specifically, exercise increased the expression of mitochondrial fusion markers while reducing the levels of fission markers, thereby restoring mitochondrial dynamic balance and ultimately mitigating myocardial injury (Ma et al., 2025b). Similarly, Jiang et al. reported that aerobic interval training elevated the expression of Mfn2 and Opa1 while decreasing Drp1 protein levels in rat myocardium, thereby improving mitochondrial function and conferring cardioprotection. Mechanistically, this effect was linked to suppression of the ERK1/2–JNK–p53 signaling pathway and upregulation of peroxisome proliferator–activated receptor gamma coactivator 1-alpha (PGC-1α) (Jiang et al., 2014).

Furthermore, myokine irisin, induced by exercise, has been shown to activate the AMPK–Nrf2 signaling axis, markedly increasing the transcription and translation of mitochondrial fusion–related genes. This promotes mitochondrial fusion, optimizes the balance of mitochondrial dynamics, and enhances mitochondrial function (Zhuo et al., 2023). Conversely, He et al. proposed that aerobic exercise may trigger mitochondrial fission and mitophagy through an FNDC5/irisin-dependent pathway. This selective clearance of damaged mitochondria improves mitochondrial quality control and cardiac function, although fusion protein levels remained unchanged in this context (He et al., 2021). The discrepancy between this study and the aforementioned findings may arise from differences in experimental focus and outcome measures rather than from conflicting biological effects. Specifically, the former emphasized mitochondrial quality control and the selective clearance of damaged mitochondria, whereas the latter primarily assessed the expression of mitochondrial fusion and fission markers and the maintenance of mitochondrial network integrity. Therefore, in models of cardiac injury, exercise may restore dynamic balance by promoting mitochondrial fusion. In contrast, in other models, the activation of fission and autophagy may be the key mechanisms for maintaining heart health and eliminating damaged mitochondria. Xiong et al. found that exhaustive exercise promotes mitochondrial fission by downregulating Mfn2 and upregulating Drp1, without significantly affecting the expression of Mfn1 and Opa1. This process significantly reduces the number of myocardial mitochondria, disrupts mitochondrial energy metabolism, and ultimately leads to myocardial injury (Xiong et al., 2022).

Exercise type and intensity are key factors in regulating myocardial mitochondrial fusion and fission processes. Studies have shown that long-term aerobic exercise promotes myocardial mitochondrial fusion and inhibits excessive fission (Fajardo et al., 2022). In contrast, a single bout of moderate-intensity acute exercise does not induce significant changes in left ventricular mitochondrial dynamics-related proteins (Yoo et al., 2019), suggesting that short-term exercise interventions may not be sufficient to trigger adaptive changes in mitochondrial morphology. Further comparisons of different exercise modalities revealed that 8 weeks of moderate-intensity interval training (60%–65% Vmax), high-intensity interval training (80%–85% Vmax), and resistance training all promoted myocardial mitochondrial fusion and inhibited pathological fission, thereby maintaining mitochondrial dynamics homeostasis, improving myocardial energy metabolism, and enhancing stress tolerance, collectively contributing to cardioprotection (Haghighi et al., 2025). However, the study also pointed out that, compared to moderate-intensity interval training, high-intensity interval training more significantly upregulated fusion-related protein expression and inhibited fission-related proteins. Although resistance training also positively influences mitochondrial dynamics, its effect was not as pronounced as that of high-intensity interval training. These results suggest that exercise intensity may be a more critical factor than exercise type in improving myocardial mitochondrial dynamics, with high-intensity interval training potentially being more effective in regulating myocardial mitochondrial dynamics.

Taken together, these findings suggest that exercise exerts cardioprotective effects by fine-tuning the balance between mitochondrial fusion and fission. However, the precise molecular mechanisms remain incompletely understood, and further research is needed to identify the exact targets and signaling pathways through which exercise regulates mitochondrial dynamics.

### 4.3 Mitochondrial calcium homeostasis in exercise-induced cardio protection

Exercise, as an easily applicable cardioprotective strategy with minimal adverse effects, plays a pivotal role in regulating Ca^2+^ homeostasis between the cytosol and mitochondria, thereby alleviating exercise deficiency–induced Ca^2+^ dysregulation and promoting cardiac health (Wei et al., 2025). Importantly, given the dynamic balance between cytosolic and mitochondrial Ca^2+^, exercise modulates the expression of key Ca^2+^-handling proteins, thereby regulating cytosolic Ca^2+^ homeostasis and indirectly contributing to the physiological control of mitochondrial Ca^2+^ concentrations (Wei et al., 2025).

Endurance exercise has been shown to enhance myocardial Ca^2+^ handling capacity in rats with heart failure induced by aortic constriction by upregulating sarcoplasmic/endoplasmic reticulum Ca^2+^-ATPase 2a (SERCA2a) and the sodium–calcium exchanger (NCX), thus improving Ca^2+^ homeostasis and exerting cardioprotective effects (da Silva et al., 2023). Starnes et al. further reported that exercise augments mitochondrial ATP production, which provides sufficient energy for SERCA2a and plasma membrane Ca^2+^ pumps to facilitate Ca^2+^ reuptake, while activation of Na^+^/K^+^-ATPase reduces the reverse driving force of NCX (Starnes and Taylor, 2007). Together, these adaptations attenuate Ca^2+^ overload and preserve cardiomyocyte Ca^2+^ homeostasis.

Exercise has also been found to stabilize mitochondrial Ca^2+^ levels, preventing Ca^2+^ overload and reducing the risk of mPTP opening (Marcil et al., 2006). This protective effect is associated with favorable alterations in the ratio of apoptosis-related proteins such as B-cell lymphoma-2 (Bcl-2) and Bcl-2–associated X protein (Bax), and appears to be substrate-dependent, being most evident when succinate serves as the metabolic substrate (Kwak et al., 2006). Kemi et al. demonstrated that exercise enhances myofilament sensitivity to Ca^2+^, thereby improving contractile function and contributing to cardioprotection (Kemi et al., 2004). Additionally, exercise reduces calpain activation and protein carbonylation, thereby limiting the degradation of Ca^2+^-handling proteins, maintaining Ca^2+^ homeostasis, and protecting the myocardium (French et al., 2008; French et al., 2006). Additionally, in the regulation of mitochondrial calcium homeostasis, exhaustive exercise and endurance exercise exhibit distinct effects. Exhaustive exercise leads to an increase in total mitochondrial Ca^2+^ content, causing calcium overload, which in turn induces the opening of the mPTP, promoting the release of free Ca^2+^ from the mitochondrial matrix and decreasing its concentration, ultimately impairing myocardial energy supply (Saris et al., 1993). In contrast, long-term endurance exercise upregulates the expression of SERCA2a and NCX, preventing mitochondrial calcium overload and mPTP opening, thereby helping to maintain myocardial mitochondrial calcium homeostasis (da Silva et al., 2023). Furthermore, after acute endurance exercise, the myocardial mitochondria’s ability to retain Ca^2+^ is enhanced (Yoo et al., 2019).

Collectively, these findings highlight that maintaining mitochondrial Ca^2+^ homeostasis is essential for sustaining and promoting cardiac health.

### 4.4 Mitochondrial oxidative stress in exercise-induced cardio protection

A substantial body of evidence indicates that exercise effectively regulates mitochondrial ROS generation and clearance, with several molecular mechanisms being implicated (Wei et al., 2025). Traditionally, the reduction in ROS levels has been attributed to two major mechanisms: i. enhanced activity of endogenous antioxidant enzyme systems, and ii. reduced production of ROS precursors such as superoxide (Farhat et al., 2015). Starnes et al. reported that long-term endurance training improved the antioxidant defense system in sedentary rats, leading to a 49% increase in catalase activity and ultimately conferring cardioprotection. Interestingly, catalase activity remained relatively low compared with other antioxidant enzymes, and no significant changes were observed in mitochondrial glutathione peroxidase or superoxide dismutase activities (Starnes et al., 2007). These findings suggest that the ROS-lowering effect of exercise may be more closely related to reduced superoxide production than to increased antioxidant enzyme activity.

In contrast, Tocantins et al. demonstrated that exercise promotes mitochondrial ROS–mediated activation of the Nrf2 signaling pathway, which upregulates downstream antioxidant enzymes and strengthens cardiac antioxidant capacity. Notably, this effect persisted for up to 8 weeks after cessation of training (Tocantins et al., 2023). The opposing viewpoints may arise from differences in the experimental models used in the two studies. Moreover, variations in training protocols may substantially influence the final outcomes, thereby leading to inconsistencies in the reported results. The divergent findings of these two studies highlight distinct mechanisms by which exercise regulates myocardial oxidative stress. In the former, exercise attenuates oxidative stress primarily by reducing ROS production, whereas in the latter, exercise increases ROS generation to activate the Nrf2 signaling pathway, thereby upregulating antioxidant enzyme expression and enhancing the heart’s antioxidant capacity. Exercise has also been shown to increase α-ketoglutarate levels, thereby suppressing atrial natriuretic peptide upregulation, reducing ROS generation and oxidative stress, and ultimately mediating cardioprotection (An et al., 2021). Moreover, complex I of the mitochondrial electron transport chain, a major site of ROS production, undergoes functional remodeling in response to endurance training (Okoye et al., 2023). Such specific adaptations effectively suppress myocardial mitochondrial ROS production, enhance antioxidant defenses, and improve cardiac performance (Farhat et al., 2015). Studies have also shown that aerobic exercise in diabetic mice activates an Akt-dependent signaling pathway, thereby suppressing the activity of mammalian Ste20-like kinase 1 (Mst1). Downregulation of Mst1 reduces mitochondrial ROS production and ultimately alleviates oxidative stress in the myocardium (Zhao et al., 2020). Notably, exercise intensity plays a pivotal role in regulating mitochondrial redox homeostasis. Evidence indicates that when rats undergo exhaustive exercise, excessive ROS are generated, accompanied by a marked decline in the activity of endogenous antioxidant enzymes. This imbalance compromises mitochondrial antioxidant defenses, leading to heightened oxidative stress and, ultimately, structural and functional injury to myocardial tissue (Yang et al., 2022).

Existing studies have shown that different forms of exercise have varying effects on myocardial oxidative stress regulation. Sabouri et al. reported that a 12-week exercise intervention for type 2 diabetes demonstrated that high-intensity interval training (HIIT), resistance training (ST), and their combined regimen all enhanced myocardial antioxidant defense capacity, as evidenced by increased superoxide dismutase (SOD) and glutathione peroxidase (GPx) activities, as well as overall antioxidant capacity. Further comparisons revealed that HIIT and combined training were significantly more effective in improving the body’s redox balance than resistance training alone (Sabouri et al., 2021). In a myocardial infarction model, both HIIT and aerobic exercise increased GPx and SOD activities, alleviated oxidative stress, but HIIT was more effective in enhancing GPx activity and reducing oxidative stress (Lu et al., 2015). Another study pointed out that both aerobic and resistance exercise can improve myocardial oxidative stress, but there was no significant difference between the two (Rodrigues et al., 2023). However, exercise has a bidirectional effect, and seven consecutive days of high-intensity exercise can lead to myocardial damage, accompanied by a decrease in mitochondrial antioxidant enzyme activity, suggesting that overtraining may have a detrimental effect on the myocardial antioxidant system (Gao et al., 2014). In conclusion, the existing evidence suggests that HIIT may have a greater advantage in improving myocardial mitochondrial redox imbalance.

Collectively, these studies highlight that exercise alleviates mitochondrial oxidative stress by reducing ROS production and/or enhancing ROS clearance, thereby preventing or attenuating oxidative myocardial injury.

### 4.5 Mitochondrial inflammatory response in exercise-induced cardio protection

Multiple studies have demonstrated that exercise exerts mitochondria-mediated anti-inflammatory effects, thereby alleviating chronic inflammation–induced myocardial injury associated with physical inactivity and contributing to the maintenance and improvement of normal cardiac function. Yuan et al. reported that exercise preserves normal sarcoplasmic reticulum Ca^2+^ release in cardiomyocytes, which safeguards mitochondrial Ca^2+^ homeostasis and mitigates oxidative stress. This adaptation prevents excessive ROS accumulation and suppresses activation of the nuclear factor kappa-B (NF-κB) pathway, ultimately reducing chronic inflammation and protecting the heart from damage (Yuan et al., 2025a).

Similarly, other studies have identified the NLRP3 inflammasome, localized at the mitochondria–endoplasmic reticulum membrane interface, as a critical target of exercise-induced cardioprotection (Lv et al., 2022). Exercise inhibits activation of the NLRP3 inflammasome by suppressing the upstream TXNIP/TRX/NF-κB signaling pathway. As a result, levels of pro-inflammatory mediators such as tumor necrosis factor-α (TNF-α), interleukin-6 (IL-6), and C-reactive protein (CRP) are markedly reduced, thereby alleviating mitochondrial inflammation and preserving cardiac function (Meng, 2017; Li et al., 2020).

Furthermore, exercise has been shown to activate the sirtuin 1 (SIRT1) signaling pathway, which enhances mitochondrial complex I activity, reduces electron leakage, and diminishes ROS production. This subsequently suppresses NF-κB–mediated inflammatory signaling and lowers pro-inflammatory cytokine expression, thereby attenuating myocardial inflammation induced by physical inactivity (Chen et al., 2018; Tuon et al., 2015). However, the cardioprotective effects of exercise are not limited to healthy individuals. Zhang et al. showed that, in an isoproterenol-induced model of cardiac inflammation, exercise suppresses the ROS–NLRP3 inflammasome signaling axis, thereby markedly attenuating myocardial inflammation, and this protective effect occurs independently of AMPK signaling (Zhang et al., 2023). Additionally, excessive or intense exercise significantly induces the production of pro-inflammatory factors such as TNF-α and IL-6, triggering a systemic inflammatory response. Importantly, this inflammatory response exacerbates mitochondrial ROS generation, further impairing mitochondrial function and creating a vicious cycle, ultimately leading to adverse effects on myocardial health (Bernecker et al., 2013).

At present, comparative studies investigating the effects of different exercise modalities on myocardial inflammatory responses remain limited. Research comparing the impact of swimming training, resistance training, and HIIT on diabetic cardiomyopathy has shown that all three exercise types reduce the expression of pro-inflammatory cytokines (such as IL-6, TNF-α, and NF-κB) and alleviate inflammation, with HIIT exhibiting the most pronounced improvement (Ma et al., 2025a). Similarly, in aged mouse models, both HIIT and moderate-intensity aerobic training exerted anti-inflammatory effects; however, HIIT was more effective in improving mitochondrial morphology and maintaining mitochondrial homeostasis, thereby more efficiently mitigating myocardial inflammation (Li et al., 2025b). In contrast, acute vigorous exercise can elicit several adverse responses, including inflammation. Li et al. demonstrated that acute exercise in rats increased myocardial mitochondrial ROS production, which in turn activated the NLRP3 inflammasome and triggered myocardial inflammation (Li et al., 2016). Collectively, these findings suggest that long-term exercise—particularly HIIT—can attenuate inflammatory responses by downregulating the expression of myocardial pro-inflammatory factors, whereas strenuous exercise tends to induce inflammation through the ROS/NLRP3 signaling pathway.

**FIGURE 2:**
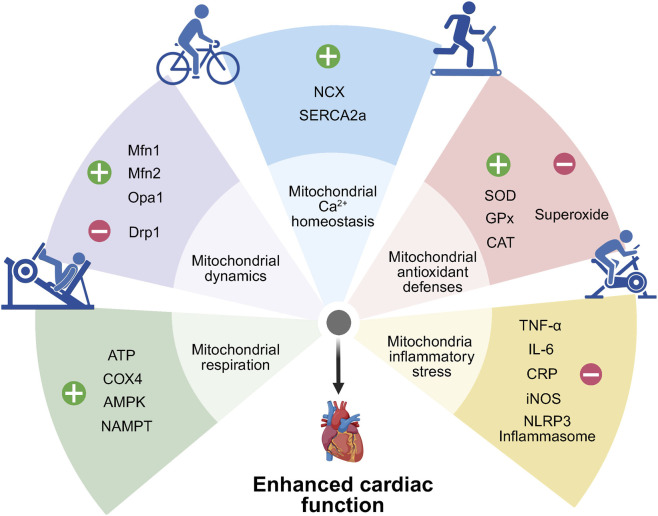
Mitochondrial mechanisms underlying exercise-induced cardioprotection. Exercise enhances cardiac protection by improving mitochondrial respiratory function, dynamics, and Ca^2+^ homeostasis, while reducing oxidative stress and inflammation. ATP, adenosine triphosphate; COX4, cytochrome c oxidase subunit 4; AMPK, AMP-activated protein kinase; NAMPT, nicotinamide phosphoribosyltransferase; Mfn1, mitofusin 1; Mfn2, mitofusin 2; Opa1, optic atrophy protein 1; Drp1, dynamin-related protein 1; NCX, sodium–calcium exchanger; SERCA2a, sarcoplasmic/endoplasmic reticulum Ca^2+^-ATPase 2a; SOD, superoxide dismutase; GPx, glutathione peroxidase; CAT, catalase; TNF-α, tumor necrosis factor-α; IL-6, interleukin-6; CRP, C-reactive protein; iNOS, inducible nitric oxide synthase; NLRP3 inflammasome, NOD-like receptor family pyrin domain–containing 3 inflammasome.

In summary, exercise optimizes mitochondrial function to downregulate chronic low-grade inflammation, thereby counteracting the risk of myocardial injury associated with sedentary behavior (Figure 2; Tables 1, 2).

**TABLE 1:** Mitochondrial mechanisms of exercise-induced cardioprotection.

Number	Subject	Indicator	Results	References
[1]	rats	ATP↑	Mitochondrial respiration↑	Lopaschuk et al. (2010)
[2]	rats	ATP↑	Mitochondrial respiration↑	Burelle et al. (2004)
[3]	humans	NO↑	Mitochondrial respiration↑	Suvorava and Cortese-Krott (2018)
[4]	mice	COX4↑, NAMPT↑, AMPK↑, mitochondrial Complex V↑	Mitochondrial respiration↑	Liang et al. (2024)
[5]	mice	Mitochondrial complex I ↑, cardiolipin↑	Mitochondrial respiration↑	Perera et al. (2025)
[6]	rats	Mfn1↑, Mfn2↑, Opa1↑, Drp1↓	Mitochondrial dynamics↑	Haghighi et al. (2025)
[7]	rats	Mfn2↑, Opa1↑, Drp1↓	Mitochondrial dynamics↑	Jiang et al. (2014)
[8]	mice	Mfn↑, Mfn2↑, Opa1↑	Mitochondrial dynamics↑	Zhuo et al. (2023)
[9]	mice	Drp1↑	Mitochondrial dynamics↑	He et al. (2021)
[10]	rats	Mfn↑, Mfn2↑, Opa1↑, Drp1↓	Mitochondrial dynamics↑	Haghighi et al. (2025)
[11]	rats	SERCA2a↑, NCX↑	Mitochondrial calcium homeostasis↑	da Silva et al. (2023)
[12]	humans	Ca^2+^ overload↓	Mitochondrial calcium homeostasis↑	Starnes and Taylor (2007)
[13]	rats	Ca^2+^ overload↓	Mitochondrial calcium homeostasis↑	Marcil et al. (2006)
[14]	rats	Myofilament Ca^2+^ sensitivity in cardiomyocytes↑	Mitochondrial calcium homeostasis↑	Kemi et al. (2004)
[15]	rats, humans	Ca^2+^ overload↑	Mitochondrial calcium homeostasis↓	Saris et al. (1993)
[16]	rats	SERCA2a↑, NCX↑	Mitochondrial calcium homeostasis↑	da Silva et al. (2023)
[17]	rats	Degradation of calcium-handling proteins↓	Mitochondrial calcium homeostasis↑	French et al. (2006)
[18]	rats	Catalase activity↑	Mitochondrial oxidative stress↓	Starnes et al. (2007)
[19]	rats	Antioxidant enzymes↑	Mitochondrial oxidative stress↓	Tocantins et al. (2023)
[20]	mice	ROS↓	Mitochondrial oxidative stress↓	An et al. (2021)
[21]	rats	ROS↓	Mitochondrial oxidative stress↓	Farhat et al. (2015)
[22]	mice	ROS↓	Mitochondrial oxidative stress↓	Zhao et al. (2020)
[23]	humans	SOD ↑, GPx ↑, TAC↑	Mitochondrial oxidative stress↓	Sabouri et al. (2021)
[24]	humans	NF-κB↓	Mitochondrial inflammatory response↓	Yuan et al. (2025a)
[25]	rats	NLRP3 inflammasome↓, TNF-α↓, IL-6↓, CRP↓	Mitochondrial inflammatory response↓	Li et al. (2020)
[26]	rats, humans	NF-κB↓	Mitochondrial inflammatory response↓	Chen et al. (2022)
[27]	mice	NLRP3 inflammasome↓	Mitochondrial inflammatory response↓	Zhang et al. (2023)

Abbreviations: ATP, adenosine triphosphate; NO, nitric oxide; COX4, cytochrome c oxidase subunit 4; NAMPT, nicotinamide phosphoribosyltransferase; AMPK, AMP-activated protein kinase; Mfn1, mitofusin 1; Mfn2, mitofusin 2; Opa1, optic atrophy protein 1; Drp1, dynamin-related protein 1; SERCA2a, sarcoplasmic/endoplasmic reticulum Ca^2+^-ATPase, 2a; NCX, sodium–calcium exchanger; ROS, reactive oxygen species; SOD, superoxide dismutase; GPx, glutathione peroxidase; TAC, total antioxidant capacity; NF-κB, Nuclear factor kappa-B; NLRP3, NOD-like receptor thermal protein domain associated protein 3; TNF-α, tumor necrosis factor-α; IL-6, interleukin-6; CRP, C-reactive protein.

**TABLE 2:** Summary of main characteristics of myocardial mitochondria under sedentary versus exercise conditions.

Main characteristics	Sedentary myocardial mitochondria	Exercise myocardial mitochondria	References
Morphology			
Mitochondrial circularity index	- Low	- High	Pasmiño et al. (2024)
Mitochondrial area	- Small	- Large	Pasmiño et al. (2024)
Mitochondrial perimeter	- Short	- Long	Pasmiño et al. (2024)
Cristae	- Disordered arrangement and even fragmentation of mitochondria	- Parallel and regular arrangement	Pasmiño et al. (2024)
Outer membrane	- Unclear boundary	- Clear outline	Pasmiño et al. (2024)
Bioenergetics			
OXPHOS efficiency	- Low	- High	Huynh and Pamenter (2022), Liang et al. (2024), Perera et al. (2025)
Rates of glucose and palmitate oxidation	- Low	- High	Huynh and Pamenter (2022), Burelle et al. (2004)
ATP production	- Decrease	- Increase	Burelle et al. (2004), Huynh and Pamenter (2022)
Calcium management	- Disrupted mitochondrial Ca^2+^ homeostasis	- Maintain mitochondrial Ca^2+^ homeostasis	da Silva et al. (2023), Quindry and Michalak (2025), Kemi et al. (2004)
ROS production	- High	- Low	Kemi et al. (2004), An et al. (2021)
Antioxidant enzyme activity	- Low	- High	Starnes et al. (2007), Tocantins et al. (2023), Sabouri et al. (2021)
Inflammatory response	- Promote NLRP3 inflammasome activation	- Block NLRP3 inflammasome activation	Yuan et al., 2025a; Meng (2017), Li et al. (2020)
Mitochondrial Life Cycle			
Mitochondrial biogenesis	- Reduce	- Enhance	Pasmiño et al. (2024)
Mitochondrial fusion and fission	- Suppress fusion - Excessive fission	- Promote fusion - Suppress fission	Haghighi et al. (2025), Jiang et al. (2014), Fajardo et al. (2022)

Abbreviations: OXPHOS, oxidative phosphorylation; ATP, adenosine triphosphate; ROS, reactive oxygen species; NF-ĸB, Nuclear factor kappa-B; NLRP3, NOD-like receptor thermal protein domain associated protein.

## 5 Multilevel protective mechanisms underlying exercise-induced remodeling of cardiac mitochondrial function

Mitochondria are central to the cardioprotective effects of exercise, and their functional remodeling is driven by a series of dynamic, coordinated molecular adaptations. Accumulating evidence indicates that exercise not only regulates mitochondrial DNA (mtDNA) copy number and heteroplasmy and preserves mitochondrial proteostasis, but also reshapes mitochondrial metabolic networks and integrates bidirectional nucleus–mitochondrion signaling at both genetic and epigenetic levels. Together, these multilevel and highly coordinated adaptations promote structural and functional optimization of cardiac mitochondria, thereby establishing a molecular basis for exercise-induced cardioprotection.

### 5.1 mtDNA copy number and heteroplasmy

Studies have shown that aerobic exercise can increase mtDNA copy number, a change that is often regarded as a molecular hallmark of enhanced mitochondrial biogenesis (Jeppesen, 2020). However, knee-extensor training in healthy individuals did not produce a significant change in mtDNA copy number in the trained limb, suggesting that exercise-induced regulation of mtDNA copy number may depend on the exercise modality (Fritzen et al., 2019). Importantly, exercise influences the mitochondrial genome not only by altering mtDNA copy number, but also through dynamic regulation of mtDNA heteroplasmy and mutation burden. The relative proportion of wild-type versus mutant mtDNA copies determines tissue-level mutation load and is strongly inversely associated with mitochondrial oxidative capacity. Aerobic exercise has been reported to markedly improve oxidative capacity in patients with mtDNA mutations, thereby reducing mutation burden and mtDNA heteroplasmy and alleviating mitochondrial dysfunction driven by elevated heteroplasmy (Jeppesen, 2020).

### 5.2 Mitochondrial proteostasis

Mitochondrial proteostasis depends on endogenous chaperones and proteolytic systems to preserve structural and functional integrity, and exercise is considered an important physiological stimulus that activates this regulatory network (Chen et al., 2021). Evidence suggests that mitochondrial lon peptidase 1 (LONP1) is a key determinant of mitochondrial proteostasis in the heart. Exercise upregulates LONP1 expression, thereby enhancing its capacity to recognize and degrade oxidatively damaged proteins (Zanini et al., 2023). In parallel, LONP1 cooperates with the mitochondrial heat shock protein 70 (mtHSP70) chaperone system to facilitate proper folding of nascent or damaged proteins, further strengthening mitochondrial protein quality control and functional homeostasis (Shin et al., 2021).

Fan et al. using proteomic analyses of a cardiomyocyte-specific heat shock protein 60 (HSP60) knockout mouse model, reported that loss of HSP60 reduces global mitochondrial protein abundance by ∼20% and triggers early activation of the mitochondrial unfolded protein response (Fan et al., 2020). By contrast, exercise interventions have been shown to increase HSP60 expression, which may partially mitigate mitochondrial protein abnormalities and help maintain mitochondrial proteostasis (D'Amico et al., 2021b). Collectively, these findings support the view that exercise remodels mitochondrial proteostasis at the molecular level by modulating mitochondrial chaperone and protease systems, thereby serving as a key regulatory node in exercise-induced mitochondrial adaptation.

### 5.3 Bidirectional nucleus–mitochondrion signaling

In the heart, exercise activates multilayered molecular regulatory networks that reshape gene expression programs and promote adaptive remodeling of cardiac structure and function. Exercise-induced changes in hemodynamic load and metabolic state engage multiple signaling pathways in cardiomyocytes and coordinate transcription factors such as PGC-1α, MEF2, and NRF2 with epigenetic regulators, thereby driving beneficial adaptations in cardiac structure and performance (Jia et al., 2019). Beyond canonical transcriptional control, exercise can also reprogram the cardiac transcriptome by reshaping non-coding RNA (ncRNA) networks, conferring cardioprotective effects (Yuan et al., 2025b). In particular, sustained training markedly remodels the expression landscape of cardiac microRNAs (miRNAs) and long non-coding RNAs (lncRNAs), which act as critical post-transcriptional and/or epigenetic modulators to fine-tune gene regulatory programs in cardiomyocytes (Yuan et al., 2025b). For example, Fathi et al. reported that 14 weeks of aerobic exercise significantly upregulated myocardial miR-1 and miR-133, enhancing the transcriptional activity of growth-associated genes and ultimately promoting a physiological hypertrophy phenotype (Fathi et al., 2020). Chang et al. further showed that exercise facilitates a specific interaction between the lncRNA Mhrt and the chromatin remodeling factor Brg1, which is implicated in pathological hypertrophy; this interaction reduces Brg1 occupancy at promoters of pro-hypertrophic genes, dampens hypertrophy-related transcriptional activation, and helps prevent maladaptive cardiac remodeling (Chang and Han, 2016). Moreover, exercise-induced cardiac reprogramming is not confined to transcriptional changes but is accompanied by broader epigenetic remodeling, including alterations in DNA methylation, histone modifications, and chromatin accessibility. These epigenetic adaptations further modulate processes central to cardiac remodeling, inflammation, and oxidative stress, collectively reinforcing exercise-driven improvements in cardiac structure and function (Ghavami et al., 2022; Shi et al., 2022). Overall, exercise-induced gene-expression reprogramming plays a pivotal role in enhancing physiological cardiac performance and increasing resistance to pathological insults.

At the genetic and epigenetic levels, exercise promotes adaptive remodeling of mitochondrial function by coordinating expression programs encoded by the nuclear and mitochondrial genomes. This process relies primarily on two complementary modes of intracellular communication: anterograde and retrograde signaling (Silver et al., 2025). Anterograde signaling originates in the nucleus and acts on mitochondria by regulating transcriptional and post-transcriptional processes of nuclear-encoded mitochondrial genes, thereby shaping mitochondrial function. Retrograde signaling, in contrast, originates from mitochondria and feeds back to the nucleus and other organelles, converting mitochondrial energetic and stress cues into transcriptional reprogramming that preserves inter-organelle homeostasis (Darfarin and Pluth, 2025).

Within the anterograde pathway, exercise activates a PGC-1α–centered transcriptional network to drive expression of nuclear-encoded mitochondrial genes. This program further modulates the activity of nuclear respiratory factor 1 (NRF1) and TFAM, thereby promoting mtDNA transcription and replication and ultimately enhancing mitochondrial capacity and adaptability (Memme and Hood, 2020; Scarpulla, 2011). In the retrograde pathway, ROS generated during mitochondrial energy production serve as key signaling mediators that engage redox-sensitive regulators such as nuclear factor erythroid 2–related factor 2 (NRF2), which in turn induces nuclear antioxidant gene programs. This feedback loop supports cellular resilience to oxidative stress and helps maintain metabolic homeostasis (Kasai et al., 2020).

In addition, the mitochondria-derived peptide MOTS-c represents an important retrograde signaling effector. Under stress conditions such as exercise, MOTS-c can translocate to the nucleus via an AMPK-dependent mechanism and bind regulatory regions enriched for antioxidant response elements (AREs), including promoters of NRF2 target genes. Through this mechanism, MOTS-c enhances transcription of NRF2-responsive genes, rewires metabolic programs, and strengthens antioxidant defenses (Kim et al., 2018; Ma, 2013; Wan et al., 2023).

### 5.4 Mitochondrial metabolic reprogramming

A substantial body of evidence indicates that regular exercise induces metabolic reprogramming of cardiac mitochondria, enabling the heart to meet energetic demands imposed by different exercise modalities. For example, swim training markedly increases the activity of electron transport chain–related enzymes and citrate synthase in the mouse heart, thereby enhancing oxidative phosphorylation capacity and supporting a more efficient and adequate energy supply during exercise (Fulghum and Hill, 2018). In an *ex vivo* study using perfused mouse hearts, moderate exercise training was shown to augment both mitochondrial glucose oxidation and fatty acid oxidation in the myocardium, improving mitochondrial metabolic function and strengthening metabolic flexibility and cardioprotection (Riehle et al., 2014).

Notably, exercise intensity appears to differentially shape cardiac mitochondrial metabolic remodeling. Hafstad et al. reported that in C57BL/6J mice subjected to 10 weeks of treadmill training, a high-intensity interval protocol elicited greater improvements in myocardial mitochondrial metabolic capacity than a moderate-intensity continuous protocol. Specifically, high-intensity interval training increased glucose oxidation, reduced palmitate oxidation, and elevated myocardial citrate synthase activity, whereas the moderate-intensity protocol did not significantly alter glucose oxidation, palmitate oxidation, or citrate synthase activity (Hafstad et al., 2011). Collectively, these findings underscore exercise intensity as a key determinant of mitochondrial metabolic adaptation and suggest that high-intensity interval treadmill training may be particularly effective in driving cardiac mitochondrial metabolic reprogramming.

### 5.5 Energetic polarity

Energetic polarity refers to the spatial compartmentalization of mitochondrial energy metabolism within distinct intracellular regions, particularly in high-demand tissues such as the heart and skeletal muscle. This concept has emerged as an important theme in mitochondrial quality control research. Current evidence suggests that, under acute exercise, changes in mitochondrial quality control may arise from locally regulated mechanisms operating within different subdomains of the mitochondrial network. The underlying link is that mitochondrial quality control is tightly coupled to mitochondrial bioenergetics; thus, local fluctuations in energy availability likely help determine where quality control processes are initiated within the cell (Drake and Yan, 2019). For example, in the heart, mitochondrial Ca^2+^ uptake and release are subject to fine spatial regulation to optimize the energetic efficiency of calcium signaling (De La Fuente et al., 2018). Moreover, under energetic stress conditions such as exercise, selective mitophagy can be observed in subsets of mitochondria within the network, further supporting the spatial specificity of mitochondrial quality control (Laker et al., 2017).

Notably, the mechanisms that establish this spatial specificity remain incompletely understood. Recent work by Drake and colleagues offers a potential explanation (Drake et al., 2021). They reported that AMPK subunits can localize to the outer mitochondrial membrane in cardiac and skeletal muscle, forming a pool termed mitochondrial AMPK (mitoAMPK). Under energetic stress, AMPK activation displays pronounced spatial heterogeneity. Specifically, following electrical stimulation–induced contraction of skeletal muscle fibers, mitoAMPK activity is not uniform across the cell but instead increases markedly within discrete mitochondrial regions (Drake et al., 2021). Collectively, these findings suggest that when cells experience elevated energetic demand or stress (e.g., during exercise), the mitochondrial energy stress response is not distributed evenly throughout the cell, but is instead concentrated within specific local subcellular domains.

### 5.6 Systems genetics and multi-omics integration

Extensive work has characterized the dynamic physiological responses of multiple organs during exercise (e.g., the heart, skeletal muscle, and liver). However, the biological mechanisms by which exercise promotes health—and the underlying molecular networks—are highly complex and remain incompletely defined. Mitochondria can adapt to cell- and tissue-specific metabolic demands through processes that involve nuclear-encoded mitochondrial genes, mtDNA heteroplasmy, and metabolite-driven energy transduction (Kappler et al., 2019). Notably, across variations in exercise intensity and duration, the transition from mitochondrial stress responses to long-term adaptation is particularly intricate, and no single pathway is sufficient to fully explain these effects.

Multi-omics approaches are increasingly helping to close this knowledge gap by systematically mapping the molecular networks that mediate exercise’s health benefits. A representative effort is the Molecular Transducers of Physical Activity Consortium (MoTrPAC), which aims to build a cross-tissue, multi-omics atlas of exercise training responses (Sanford et al., 2020). Recently, MoTrPAC generated a mitochondrial multi-omics landscape across 19 distinct tissues under aerobic exercise conditions, highlighting in particular that aerobic exercise alters acetylation-related modifications of cardiac mitochondrial proteins (Amar et al., 2024). Although prior studies support the notion that exercise improves mitochondrial content and function, mitochondria-specific multi-omics signatures across different exercise modalities, intensities, and training durations remain to be more comprehensively delineated (Yan et al., 2012; Santos-Alves et al., 2015).

Beyond delineating the biological changes and mechanisms elicited by exercise, it is equally important to understand why the benefits of regular physical activity vary substantially across individuals. As early as four decades ago, researchers documented marked inter-individual variability in training responsiveness: in a 20-week aerobic training program, participants exhibited wide differences in the improvement of VO_2_max, suggesting that certain genetic factors may confer greater sensitivity to training stimuli (Lortie et al., 1984). By extension, exercise-induced mitochondrial adaptations are also likely shaped by genetic background, with candidate regulators including tumor protein p53 (TP53) and N-acetyltransferase 1 (Nat1). TP53, encoded by the human TP53 gene, is required for normal mitochondrial respiration and mtDNA integrity; its loss impairs these processes and reduces aerobic exercise capacity (Park et al., 2009). Similarly, Nat1-deficient mice display significant reductions in basal metabolic rate and exercise performance (Chennamsetty et al., 2016). Genome-wide approaches can further resolve an individual’s genetic architecture and may even identify rare variants that exert beneficial or detrimental effects on exercise responsiveness (Bouchard et al., 2015).

In summary, individual-level multi-omics profiling can integrate exercise state–dependent molecular changes with genetic information. By accounting for a person’s specific responses to different exercise modalities and intensities, these approaches provide a mechanistic foundation for developing targeted, personalized exercise prescriptions.

## 6 Roles of non-myocyte cardiac cells in exercise-induced cardioprotection

### 6.1 Fibroblasts

Exercise has been clearly shown to confer cardioprotection in part by regulating the physiological functions of cardiac fibroblasts. Fu et al. reported that regular exercise promotes the release of exosomes derived from endothelial progenitor cells, thereby increasing miR-126 expression. This, in turn, suppresses activation of the TGF-β/Smad3 signaling pathway, slows fibroblast transdifferentiation, and markedly attenuates myocardial fibrosis, ultimately protecting the heart (Fu et al., 2024).

In addition, other studies have found that exercise upregulates fibroblast growth factor 21 (FGF21) expression and inhibits activation of the TGF-β1–Smad2/3–MMP2/9 signaling axis, thereby reducing collagen production. This mechanism effectively alleviates myocardial fibrosis in mouse models of myocardial infarction and improves cardiac function (Ma et al., 2021). Collectively, these findings indicate that exercise protects the myocardium from injury by improving fibroblast physiology and suppressing fibroblast-driven fibrosis and collagen deposition.

### 6.2 Endothelial cells

Multiple lines of evidence indicate that cardiac endothelial cells serve as key mediators of exercise-induced cardioprotection, a process that engages diverse molecular signaling pathways and regulatory mechanisms. Bernardo et al. demonstrated that exercise stimulates cardiac endothelial cells to produce substantial amounts of nitric oxide (NO). This gaseous signaling molecule helps preserve cellular energy homeostasis by modulating mitochondrial respiration, thereby optimizing endothelial function, reducing cardiac stress, and ultimately promoting cardioprotection (Bernardo et al., 2018).

Studies also show that exercise markedly lowers systemic inflammation by downregulating inflammatory markers such as TNF-α and IL-6, which in turn helps prevent inflammation-driven endothelial dysfunction in the heart. This anti-inflammatory effect is important for maintaining cardiac health and reducing the risk of cardiovascular disease (Isaksen et al., 2019). Taken together, these findings underscore that preserving normal cardiac endothelial cell function is essential for sustaining overall cardiac performance.

### 6.3 Immune cells

Immune cells are not only a primary line of defense against invading pathogens but also play pivotal roles in maintaining cardiac homeostasis and orchestrating stress responses (Rurik et al., 2021). Bai et al. found that downhill running training increases the abundance of T cells, NK cells, and M2 macrophages in myocardial tissue, thereby shaping an anti-inflammatory, pro-reparative immune microenvironment. This milieu promotes repair and regenerative processes in injured myocardium, reduces myocardial fibrosis, and ultimately confers cardioprotection (Bai et al., 2025).

In addition, Wang et al. reported that running training upregulates the inhibitory receptor FcγRIIB on the surface of B cells. By raising the activation threshold of B cells, this mechanism suppresses cardiac inflammation and antibody-mediated tissue injury, thereby significantly attenuating doxorubicin (Dox)-induced cardiotoxicity (Wang et al., 2024). Collectively, these findings suggest that immune cells help preserve myocardial homeostasis—and support cardiac health—by remodeling the cardiac immune microenvironment and restraining excessive inflammatory responses.

## 7 Limitation

The current research still faces several limitations. Most studies investigating the mitochondrial mechanisms of exercise-induced cardioprotection remain at the basic experimental level, with relatively few and insufficiently in-depth clinical studies in humans. Although animal studies consistently demonstrate that exercise markedly improves mitochondrial function, the magnitude and temporal dynamics of these adaptations in humans often differ and are modulated by factors such as age, training status, and disease background. Therefore, caution is warranted when extrapolating findings from animal models directly to human exercise physiology. While preclinical studies provide important mechanistic insights, human responses to exercise are shaped by greater physiological complexity, inter-individual variability, and environmental factors. Future work should prioritize high-quality intervention trials in diverse populations to facilitate the translation of experimental findings into practical applications. In addition, although extensive evidence supports the beneficial effects of exercise on cardiac function, considerable heterogeneity exists in the exercise interventions employed across studies, and a unified standard is lacking. Moving forward, greater efforts are needed to establish both standardized and personalized exercise prescriptions, systematically optimizing training protocols to maximize the cardioprotective benefits of different exercise modalities in the general population. In addition, current studies have paid limited attention to emerging layers of mitochondrial regulation—such as the influence of age and sex on mitochondria-mediated exercise-induced cardioprotection, as well as epigenetic modifications, long non-coding RNAs, microRNAs, and acetylation-mediated control—and the key signaling pathways through which these mechanisms shape exercise-induced myocardial adaptation and cardioprotection remain to be systematically elucidated. At the same time, future studies may further incorporate emerging computational models and systematically integrate analyses of exercise adaptation–related anterograde and retrograde signaling pathways. Such approaches would enable a deeper, multiscale understanding of exercise-induced physiological regulatory mechanisms and provide a more precise and predictive theoretical framework for elucidating their underlying causal relationships.

## 8 Conclusion

This review highlights cardiac mitochondria as central targets of exercise-induced cardioprotection, summarizing regulatory effects across five domains: mitochondrial respiration, dynamics, calcium homeostasis, antioxidant defense, and inflammatory response. Numerous studies consistently indicate that exercise enhances myocardial metabolic adaptability and functional reserve by improving mitochondrial respiration, calcium homeostasis, and reducing systemic inflammation. However, the mechanisms by which exercise regulates mitochondrial dynamics and combats oxidative stress remain unclear, with existing evidence presenting contradictions. Further exploration of these potential regulatory mechanisms is therefore needed. Furthermore, the extent of exercise-induced cardioprotective effects appears to be closely influenced by exercise type, intensity, and duration.

## References

[B1] Adams R. A. Liu Z. Hsieh C. Marko M. Lederer W. J. Jafri M. S. (2023). Structural analysis of mitochondria in cardiomyocytes: insights into bioenergetics and membrane remodeling. Curr. Issues Mol. Biol. 45, 6097–6115. 10.3390/cimb45070385 37504301 PMC10378267

[B2] Adebayo M. Singh S. Singh A. P. Dasgupta S. (2021). Mitochondrial fusion and fission: the fine-tune balance for cellular homeostasis. Faseb J. 35, e21620. 10.1096/fj.202100067R 34048084 PMC8415099

[B3] Amar D. Gay N. R. Jimenez-Morales D. Jean Beltran P. M. Ramaker M. E. Raja A. N. (2024). The mitochondrial multi-omic response to exercise training across rat tissues. Cell Metab. 36, 1411–1429.e10. 10.1016/j.cmet.2023.12.021 38701776 PMC11152996

[B4] An D. Zeng Q. Zhang P. Ma Z. Zhang H. Liu Z. (2021). Alpha-ketoglutarate ameliorates pressure overload-induced chronic cardiac dysfunction in mice. Redox Biol. 46, 102088. 10.1016/j.redox.2021.102088 34364218 PMC8353361

[B5] Andrieux P. Chevillard C. Cunha-Neto E. Nunes J. P. S. (2021). Mitochondria as a cellular hub in infection and inflammation. Int. J. Mol. Sci. 22, 11338. 10.3390/ijms222111338 34768767 PMC8583510

[B6] Arslan F. de Kleijn D. P. Pasterkamp G. (2011). Innate immune signaling in cardiac ischemia. Nat. Rev. Cardiol. 8, 292–300. 10.1038/nrcardio.2011.38 21448140

[B7] Atici A. E. Crother T. R. Noval Rivas M. (2023). Mitochondrial quality control in health and cardiovascular diseases. Front. Cell Dev. Biol. 11, 1290046. 10.3389/fcell.2023.1290046 38020895 PMC10657886

[B8] Bai X. Zhang W. Zhang Y. Xu X. (2025). Downhill running regulates cardiac immune response through GCN2. PLoS One 20, e0329973. 10.1371/journal.pone.0329973 40845009 PMC12373192

[B9] Banerjee I. Fuseler J. W. Price R. L. Borg T. K. Baudino T. A. (2007). Determination of cell types and numbers during cardiac development in the neonatal and adult rat and mouse. Am. J. Physiol. Heart Circ. Physiol. 293, H1883–H1891. 10.1152/ajpheart.00514.2007 17604329

[B10] Bernardo B. C. Ooi J. Y. Y. Weeks K. L. Patterson N. L. Mcmullen J. R. (2018). Understanding key mechanisms of exercise-induced cardiac protection to mitigate disease: current knowledge and emerging concepts. Physiol. Rev. 98, 419–475. 10.1152/physrev.00043.2016 29351515

[B11] Bernecker C. Scherr J. Schinner S. Braun S. Scherbaum W. A. Halle M. (2013). Evidence for an exercise induced increase of TNF-α and IL-6 in marathon runners. Scand. J. Med. Sci. Sports 23, 207–214. 10.1111/j.1600-0838.2011.01372.x 22092703

[B12] Bishop D. J. Lee M. J. Picard M. (2025). Exercise as mitochondrial medicine: how does the exercise prescription affect mitochondrial adaptations to training? Annu. Rev. Physiol. 87, 107–129. 10.1146/annurev-physiol-022724-104836 39656953

[B13] Bouchard C. Antunes-Correa L. M. Ashley E. A. Franklin N. Hwang P. M. Mattsson C. M. (2015). Personalized preventive medicine: genetics and the response to regular exercise in preventive interventions. Prog. Cardiovasc Dis. 57, 337–346. 10.1016/j.pcad.2014.08.005 25559061 PMC4285566

[B14] Bulthuis E. P. Dieteren C. E. J. Bergmans J. Berkhout J. Wagenaars J. A. van de Westerlo E. M. A. (2023). Stress-dependent macromolecular crowding in the mitochondrial matrix. Embo J. 42, e108533. 10.15252/embj.2021108533 36825437 PMC10068333

[B15] Burelle Y. Wambolt R. B. Grist M. Parsons H. L. Chow J. C. Antler C. (2004). Regular exercise is associated with a protective metabolic phenotype in the rat heart. Am. J. Physiol. Heart Circ. Physiol. 287, H1055–H1063. 10.1152/ajpheart.00925.2003 15105170

[B16] Campos J. C. Marchesi Bozi L. H. Krum B. Grassmann Bechara L. R. Ferreira N. D. Arini G. S. (2023). Exercise preserves physical fitness during aging through AMPK and mitochondrial dynamics. Proc. Natl. Acad. Sci. U. S. A. 120, e2204750120. 10.1073/pnas.2204750120 36595699 PMC9926278

[B17] Chaanine A. H. Joyce L. D. Stulak J. M. Maltais S. Joyce D. L. Dearani J. A. (2019). Mitochondrial morphology, dynamics, and function in human pressure overload or ischemic heart disease with preserved or reduced ejection fraction. Circ. Heart Fail 12, e005131. 10.1161/circheartfailure.118.005131 30744415

[B18] Chan D. C. (2020). Mitochondrial dynamics and its involvement in disease. Annu. Rev. Pathol. 15, 235–259. 10.1146/annurev-pathmechdis-012419-032711 31585519

[B19] Chang C. P. Han P. (2016). Epigenetic and lncRNA regulation of cardiac pathophysiology. Biochim. Biophys. Acta 1863, 1767–1771. 10.1016/j.bbamcr.2016.03.005 26969820 PMC7393981

[B20] Chen W. K. Tsai Y. L. Shibu M. A. Shen C. Y. Chang-Lee S. N. Chen R. J. (2018). Exercise training augments Sirt1-signaling and attenuates cardiac inflammation in D-galactose induced-aging rats. Aging (Albany NY) 10, 4166–4174. 10.18632/aging.101714 30582744 PMC6326662

[B21] Chen Z. Huang L. Tso A. Wang S. Fang X. Ouyang K. (2021). Mitochondrial chaperones and proteases in cardiomyocytes and heart failure. Front. Mol. Biosci. 8, 630332. 10.3389/fmolb.2021.630332 33937324 PMC8082175

[B22] Chen H. Chen C. Spanos M. Li G. Lu R. Bei Y. (2022). Exercise training maintains cardiovascular health: signaling pathways involved and potential therapeutics. Signal Transduct. Target Ther. 7, 306. 10.1038/s41392-022-01153-1 36050310 PMC9437103

[B23] Chen W. Zhao H. Li Y. (2023). Mitochondrial dynamics in health and disease: mechanisms and potential targets. Signal Transduct. Target Ther. 8, 333. 10.1038/s41392-023-01547-9 37669960 PMC10480456

[B24] Chennamsetty I. Coronado M. Contrepois K. Keller M. P. Carcamo-Orive I. Sandin J. (2016). Nat1 deficiency is associated with mitochondrial dysfunction and exercise intolerance in mice. Cell Rep. 17, 527–540. 10.1016/j.celrep.2016.09.005 27705799 PMC5097870

[B25] D'Amico D. Andreux P. A. ValdéS P. Singh A. Rinsch C. Auwerx J. (2021a). Impact of the natural compound urolithin A on health, disease, and aging. Trends Mol. Med. 27, 687–699. 10.1016/j.molmed.2021.04.009 34030963

[B26] D'Amico D. Fiore R. Caporossi D. di Felice V. D. Cappello F. Dimauro I. (2021b). Function and fiber-type specific distribution of Hsp60 and αB-Crystallin in skeletal muscles: role of physical exercise. Biol. (Basel) 10, 77. 10.3390/biology10020077 PMC7911561 33494467

[B27] Da Silva V. L. Mota G. A. F. de Souza S. L. B. de Campos D. H. S. Melo A. B. Vileigas D. F. (2023). Aerobic exercise training improves calcium handling and cardiac function in rats with heart failure resulting from aortic stenosis. Int. J. Mol. Sci. 24, 12306. 10.3390/ijms241512306 37569680 PMC10418739

[B28] Darfarin G. Pluth J. (2025). Mitochondria-nuclear crosstalk: orchestrating mtDNA maintenance. Environ. Mol. Mutagen 66, 222–242. 10.1002/em.70013 40418056 PMC12235075

[B29] de La Fuente S. Lambert J. P. Nichtova Z. Fernandez Sanz C. Elrod J. W. Sheu S. S. (2018). Spatial separation of mitochondrial calcium uptake and extrusion for energy-efficient mitochondrial calcium signaling in the heart. Cell Rep. 24, 3099–3107.e4. 10.1016/j.celrep.2018.08.040 30231993 PMC6226263

[B30] Ding P. Song Y. Yang Y. Zeng C. (2024). NLRP3 inflammasome and pyroptosis in cardiovascular diseases and exercise intervention. Front. Pharmacol. 15, 1368835. 10.3389/fphar.2024.1368835 38681198 PMC11045953

[B31] Disatnik M. H. Ferreira J. C. Campos J. C. Gomes K. S. Dourado P. M. Qi X. (2013). Acute inhibition of excessive mitochondrial fission after myocardial infarction prevents long-term cardiac dysfunction. J. Am. Heart Assoc. 2, e000461. 10.1161/jaha.113.000461 24103571 PMC3835263

[B32] Dos Santos J. A. C. Veras A. S. C. Batista V. R. G. Tavares M. E. A. Correia R. R. Suggett C. B. (2022). Physical exercise and the functions of microRNAs. Life Sci. 304, 120723. 10.1016/j.lfs.2022.120723 35718233

[B33] Drake J. C. Yan Z. (2019). Precision remodeling: how exercise improves mitochondrial quality in myofibers. Curr. Opin. Physiol. 10, 96–101. 10.1016/j.cophys.2019.05.005 32832743 PMC7434048

[B34] Drake J. C. Wilson R. J. Laker R. C. Guan Y. Spaulding H. R. Nichenko A. S. (2021). Mitochondria-localized AMPK responds to local energetics and contributes to exercise and energetic stress-induced mitophagy. Proc. Natl. Acad. Sci. U. S. A. 118, e2025932118. 10.1073/pnas.2025932118 34493662 PMC8449344

[B35] Duan C. Kuang L. Hong C. Xiang X. Liu J. Li Q. (2021). Mitochondrial Drp1 recognizes and induces excessive mPTP opening after hypoxia through BAX-PiC and LRRK2-HK2. Cell Death Dis. 12, 1050. 10.1038/s41419-021-04343-x 34741026 PMC8571301

[B36] Dubois M. Pallot F. Gouin-Gravezat M. Boulghobra D. Coste F. Walther G. (2024). Exercise training May reduce fragmented mitochondria in the ischemic-reperfused heart through DRP1. J. Gen. Physiol. 156, e202313485. 10.1085/jgp.202313485 39508760 PMC11551008

[B37] Fajardo G. Coronado M. Matthews M. Bernstein D. (2022). Mitochondrial quality control in the heart: the balance between physiological and pathological stress. Biomedicines 10, 1375. 10.3390/biomedicines10061375 35740401 PMC9220167

[B38] Fan H. He Z. Huang H. Zhuang H. Liu H. Liu X. (2020). Mitochondrial quality control in cardiomyocytes: a critical role in the progression of cardiovascular diseases. Front. Physiol. 11, 252. 10.3389/fphys.2020.00252 32292354 PMC7119225

[B39] Farhat F. Dupas J. Amérand A. Goanvec C. Feray A. Simon B. (2015). Effect of exercise training on oxidative stress and mitochondrial function in rat heart and gastrocnemius muscle. Redox Rep. 20, 60–68. 10.1179/1351000214y.0000000105 25242065 PMC6837578

[B40] Fathi M. Gharakhanlou R. Rezaei R. (2020). The changes of heart miR-1 and miR-133 expressions following physiological hypertrophy due to endurance training. Cell J. 22, 133–140. 10.22074/cellj.2020.7014 32779443 PMC7481891

[B41] Feng N. Yu H. Wang Y. Zhang Y. Xiao H. Gao W. (2023). Exercise training attenuates angiotensin II-induced cardiac fibrosis by reducing POU2F1 expression. J. Sport Health Sci. 12, 464–476. 10.1016/j.jshs.2022.10.004 36374849 PMC10362488

[B42] French J. P. Quindry J. C. Falk D. J. Staib J. L. Lee Y. Wang K. K. (2006). Ischemia-reperfusion-induced calpain activation and SERCA2a degradation are attenuated by exercise training and calpain inhibition. Am. J. Physiol. Heart Circ. Physiol. 290, H128–H136. 10.1152/ajpheart.00739.2005 16155100

[B43] French J. P. Hamilton K. L. Quindry J. C. Lee Y. Upchurch P. A. Powers S. K. (2008). Exercise-induced protection against myocardial apoptosis and necrosis: mnsod, calcium-handling proteins, and calpain. Faseb J. 22, 2862–2871. 10.1096/fj.07-102541 18417547 PMC2493460

[B44] Fritzen A. M. Thøgersen F. B. Thybo K. Vissing C. R. Krag T. O. Ruiz-Ruiz C. (2019). Adaptations in mitochondrial enzymatic activity occurs independent of genomic dosage in response to aerobic exercise training and deconditioning in human skeletal muscle. Cells 8, 237. 10.3390/cells8030237 30871120 PMC6468422

[B45] Fu G. Wang Z. Hu S. (2024). Exercise improves cardiac fibrosis by stimulating the release of endothelial progenitor cell-derived exosomes and upregulating miR-126 expression. Front. Cardiovasc Med. 11, 1323329. 10.3389/fcvm.2024.1323329 38798919 PMC11119291

[B46] Fulghum K. Hill B. G. (2018). Metabolic mechanisms of exercise-induced cardiac remodeling. Front. Cardiovasc Med. 5, 127. 10.3389/fcvm.2018.00127 30255026 PMC6141631

[B47] Gandre-Babbe S. van der Bliek A. M. (2008). The novel tail-anchored membrane protein Mff controls mitochondrial and peroxisomal fission in mammalian cells. Mol. Biol. Cell 19, 2402–2412. 10.1091/mbc.e07-12-1287 18353969 PMC2397315

[B48] Gao S. Hu J. (2021). Mitochondrial fusion: the machineries in and out. Trends Cell Biol. 31, 62–74. 10.1016/j.tcb.2020.09.008 33092941

[B49] Gao C. Chen X. Li J. Li Y. Tang Y. Liu L. (2014). Myocardial mitochondrial oxidative stress and dysfunction in intense exercise: regulatory effects of quercetin. Eur. J. Appl. Physiol. 114, 695–705. 10.1007/s00421-013-2802-9 24368555

[B50] Ghavami S. Zamani M. Ahmadi M. Erfani M. Dastghaib S. Darbandi M. (2022). Epigenetic regulation of autophagy in gastrointestinal cancers. Biochim. Biophys. Acta Mol. Basis Dis. 1868, 166512. 10.1016/j.bbadis.2022.166512 35931405

[B51] Ghezzi D. Zeviani M. (2018). Human diseases associated with defects in assembly of OXPHOS complexes. Essays Biochem. 62, 271–286. 10.1042/ebc20170099 30030362 PMC6056716

[B52] Gibb A. A. Hill B. G. (2018). Metabolic coordination of physiological and pathological cardiac remodeling. Circ. Res. 123, 107–128. 10.1161/circresaha.118.312017 29929976 PMC6023588

[B53] Godoy Coto J. Pereyra E. V. Cavalli F. A. Valverde C. A. Caldiz C. I. Maté S. M. (2024). Exercise-induced cardiac mitochondrial reorganization and enhancement in spontaneously hypertensive rats. Pflugers Arch. 476, 1109–1123. 10.1007/s00424-024-02956-7 38625371

[B54] Goetzman E. Gong Z. Zhang B. Muzumdar R. (2023). Complex II biology in aging, health, and disease. Antioxidants (Basel) 12, 1477. 10.3390/antiox12071477 37508015 PMC10376733

[B55] Granata C. Oliveira R. S. Little J. P. Renner K. Bishop D. J. (2016). Training intensity modulates changes in PGC-1α and p53 protein content and mitochondrial respiration, but not markers of mitochondrial content in human skeletal muscle. Faseb J. 30, 959–970. 10.1096/fj.15-276907 26572168

[B56] Granata C. Jamnick N. A. Bishop D. J. (2018). Training-induced changes in mitochondrial content and respiratory function in human skeletal muscle. Sports Med. 48, 1809–1828. 10.1007/s40279-018-0936-y 29934848

[B57] Guan S. Zhao L. Peng R. (2022). Mitochondrial respiratory chain supercomplexes: from structure to function. Int. J. Mol. Sci. 23, 13880. 10.3390/ijms232213880 36430359 PMC9696846

[B58] Gupta A. Becker T. (2021). Mechanisms and pathways of mitochondrial outer membrane protein biogenesis. Biochim. Biophys. Acta Bioenerg. 1862, 148323. 10.1016/j.bbabio.2020.148323 33035511

[B59] Hafstad A. D. Boardman N. T. Lund J. Hagve M. Khalid A. M. WisløFF U. (2011). High intensity interval training alters substrate utilization and reduces oxygen consumption in the heart. J. Appl. Physiol. (1985) 111, 1235–1241. 10.1152/japplphysiol.00594.2011 21836050

[B60] Haghighi A. H. Bandali M. R. Askari R. Shahrabadi H. Barone R. Bei R. (2025). The effects of different exercise training protocols on mitochondrial dynamics in skeletal and cardiac muscles of wistar rats. J. Orthop. Surg. Res. 20, 395. 10.1186/s13018-025-05809-w 40251584 PMC12008994

[B61] Hasan P. Berezhnaya E. RodríGUEZ-Prados M. Weaver D. Bekeova C. Cartes-Saavedra B. (2024). MICU1 and MICU2 control mitochondrial calcium signaling in the mammalian heart. Proc. Natl. Acad. Sci. U. S. A. 121, e2402491121. 10.1073/pnas.2402491121 39163336 PMC11363308

[B62] He W. Tang Y. Li C. Zhang X. Huang S. Tan B. (2021). Exercise enhanced cardiac function in mice with radiation-induced heart disease *via* the FNDC5/Irisin-Dependent mitochondrial turnover pathway. Front. Physiol. 12, 739485. 10.3389/fphys.2021.739485 34899376 PMC8660102

[B63] Hernandez-Resendiz S. Prakash A. Loo S. J. Semenzato M. Chinda K. Crespo-Avilan G. E. (2023). Targeting mitochondrial shape: at the heart of cardioprotection. Basic Res. Cardiol. 118, 49. 10.1007/s00395-023-01019-9 37955687 PMC10643419

[B64] Hinton A. JR. Claypool S. M. Neikirk K. Senoo N. Wanjalla C. N. Kirabo A. (2024). Mitochondrial structure and function in human heart failure. Circ. Res. 135, 372–396. 10.1161/circresaha.124.323800 38963864 PMC11225798

[B65] Huang Z. Wilson J. J. (2023). Structure-activity relationships of metal-based inhibitors of the mitochondrial calcium uniporter. ChemMedChem 18, e202300106. 10.1002/cmdc.202300106 37015871

[B66] Huynh K. W. Pamenter M. E. (2022). Lactate inhibits naked mole-rat cardiac mitochondrial respiration. J. Comp. Physiol. B 192, 501–511. 10.1007/s00360-022-01430-z 35181821

[B67] Isaksen K. Halvorsen B. Munk P. S. Aukrust P. Larsen A. I. (2019). Effects of interval training on inflammatory biomarkers in patients with ischemic heart failure. Scand. Cardiovasc J. 53, 213–219. 10.1080/14017431.2019.1629004 31169417

[B68] Isei M. O. Stevens D. Kamunde C. (2021). Temperature rise and copper exposure reduce heart mitochondrial reactive oxygen species scavenging capacity. Comp. Biochem. Physiol. C Toxicol. Pharmacol. 243, 108999. 10.1016/j.cbpc.2021.108999 33556536

[B69] Jeppesen T. D. (2020). Aerobic exercise training in patients with mtDNA-Related mitochondrial myopathy. Front. Physiol. 11, 349. 10.3389/fphys.2020.00349 32508662 PMC7253634

[B70] Jia D. Hou L. Lv Y. Xi L. Tian Z. (2019). Postinfarction exercise training alleviates cardiac dysfunction and adverse remodeling *via* mitochondrial biogenesis and SIRT1/PGC-1α/PI3K/Akt signaling. J. Cell Physiol. 234, 23705–23718. 10.1002/jcp.28939 31187505

[B71] Jiang H. K. Wang Y. H. Sun L. He X. Zhao M. Feng Z. H. (2014). Aerobic interval training attenuates mitochondrial dysfunction in rats post-myocardial infarction: roles of mitochondrial network dynamics. Int. J. Mol. Sci. 15, 5304–5322. 10.3390/ijms15045304 24675698 PMC4013565

[B72] Jin J. Y. Wei X. X. Zhi X. L. Wang X. H. Meng D. (2021). Drp1-dependent mitochondrial fission in cardiovascular disease. Acta Pharmacol. Sin. 42, 655–664. 10.1038/s41401-020-00518-y 32913266 PMC8115655

[B73] Kappler L. Hoene M. Hu C. von Toerne C. Li J. Bleher D. (2019). Linking bioenergetic function of mitochondria to tissue-specific molecular fingerprints. Am. J. Physiol. Endocrinol. Metab. 317, E374–e387. 10.1152/ajpendo.00088.2019 31211616

[B74] Kasai S. Shimizu S. Tatara Y. Mimura J. Itoh K. (2020). Regulation of Nrf2 by mitochondrial reactive oxygen species in physiology and pathology. Biomolecules 10, 320. 10.3390/biom10020320 32079324 PMC7072240

[B75] Kemi O. J. Haram P. M. WisløFF U. Ellingsen Ø. (2004). Aerobic fitness is associated with cardiomyocyte contractile capacity and endothelial function in exercise training and detraining. Circulation 109, 2897–2904. 10.1161/01.Cir.0000129308.04757.72 15173028

[B76] Kim K. H. Son J. M. Benayoun B. A. Lee C. (2018). The mitochondrial-encoded peptide MOTS-c translocates to the nucleus to regulate nuclear gene expression in response to metabolic stress. Cell Metab. 28, 516–524.e7. 10.1016/j.cmet.2018.06.008 29983246 PMC6185997

[B77] Kwak H. B. Song W. Lawler J. M. (2006). Exercise training attenuates age-induced elevation in Bax/Bcl-2 ratio, apoptosis, and remodeling in the rat heart. Faseb J. 20, 791–793. 10.1096/fj.05-5116fje 16459353

[B78] Laker R. C. Drake J. C. Wilson R. J. Lira V. A. Lewellen B. M. Ryall K. A. (2017). Ampk phosphorylation of Ulk1 is required for targeting of mitochondria to lysosomes in exercise-induced mitophagy. Nat. Commun. 8, 548. 10.1038/s41467-017-00520-9 28916822 PMC5601463

[B79] Laranjo L. Lanas F. Sun M. C. Chen D. A. Hynes L. Imran T. F. (2024). World heart Federation roadmap for secondary prevention of cardiovascular disease: 2023 update. Glob. Heart 19, 8. 10.5334/gh.1278 38273995 PMC10809857

[B80] Lee Y. J. Jeong S. Y. Karbowski M. Smith C. L. Youle R. J. (2004). Roles of the mammalian mitochondrial fission and fusion mediators Fis1, Drp1, and Opa1 in apoptosis. Mol. Biol. Cell 15, 5001–5011. 10.1091/mbc.e04-04-0294 15356267 PMC524759

[B81] Li H. Miao W. Ma J. XV Z. Bo H. Li J. (2016). Acute exercise-induced mitochondrial stress triggers an inflammatory response in the myocardium *via* NLRP3 inflammasome activation with mitophagy. Oxid. Med. Cell Longev. 2016, 1987149. 10.1155/2016/1987149 26770647 PMC4684864

[B82] Li Y. Xu P. Wang Y. Zhang J. Yang M. Chang Y. (2020). Different intensity exercise preconditions affect cardiac function of exhausted rats through regulating TXNIP/TRX/NF-ĸB(p65)/NLRP3 inflammatory pathways. Evid. Based Complement. Altern. Med. 2020, 5809298. 10.1155/2020/5809298 32595731 PMC7301185

[B83] Li J. J. Ping Z. Wang Z. W. Wang Y. B. Shi C. M. Cao X. B. (2022). Experimental study of mitochondrion-targeted small molecule IR-61 ameliorated exhaustive exercise-induced cardiac injury in rats. Zhongguo Ying Yong Sheng Li Xue Za Zhi 38, 497–503. 10.12047/j.cjap.6260.2022.093 37088759

[B84] Li K. Li S. Jia H. Song Y. Chen Z. Wang Y. (2025a). Aerobic exercise alleviates cardiac dysfunction correlated with lipidomics and mitochondrial quality control. Antioxidants (Basel) 14, 748. 10.3390/antiox14060748 40563380 PMC12189445

[B85] Li Q. Liu Q. Lin Z. Lin W. Lin Z. Huang F. (2025b). Comparison between the effect of mid-late-life high-intensity interval training and continuous moderate-intensity training in old mouse hearts. J. Gerontol. A Biol. Sci. Med. Sci. 80, glaf025. 10.1093/gerona/glaf025 39928548 PMC11973967

[B86] Liang R. Hou X. Zhou D. Zhu L. Teng L. Song W. (2024). Exercise preconditioning mitigates ischemia-reperfusion injury in rats by enhancing mitochondrial respiration. Neuroscience 562, 64–74. 10.1016/j.neuroscience.2024.10.045 39461659

[B87] Liu S. Lai J. Feng Y. Zhuo Y. Zhang H. Chen Y. (2023a). Acetyl-CoA carboxylase 1 depletion suppresses *de novo* fatty acid synthesis and mitochondrial β-oxidation in castration-resistant prostate cancer cells. J. Biol. Chem. 299, 102720. 10.1016/j.jbc.2022.102720 36410440 PMC9771725

[B88] Liu Z. Y. Song K. Tu B. Lin L. C. Sun H. Zhou Y. (2023b). Crosstalk between oxidative stress and epigenetic marks: new roles and therapeutic implications in cardiac fibrosis. Redox Biol. 65, 102820. 10.1016/j.redox.2023.102820 37482041 PMC10369469

[B89] Lopaschuk G. D. Ussher J. R. Folmes C. D. Jaswal J. S. Stanley W. C. (2010). Myocardial fatty acid metabolism in health and disease. Physiol. Rev. 90, 207–258. 10.1152/physrev.00015.2009 20086077

[B90] Lopaschuk G. D. Karwi Q. G. Tian R. Wende A. R. Abel E. D. (2021). Cardiac energy metabolism in heart failure. Circ. Res. 128, 1487–1513. 10.1161/circresaha.121.318241 33983836 PMC8136750

[B91] Lortie G. Simoneau J. A. Hamel P. Boulay M. R. Landry F. Bouchard C. (1984). Responses of maximal aerobic power and capacity to aerobic training. Int. J. Sports Med. 5, 232–236. 10.1055/s-2008-1025911 6500788

[B92] Lu K. Wang L. Wang C. Yang Y. Hu D. Ding R. (2015). Effects of high-intensity interval *versus* continuous moderate-intensity aerobic exercise on apoptosis, oxidative stress and metabolism of the infarcted myocardium in a rat model. Mol. Med. Rep. 12, 2374–2382. 10.3892/mmr.2015.3669 25936391

[B93] Lu X. Thai P. N. Lu S. Pu J. Bers D. M. (2019). Intrafibrillar and perinuclear mitochondrial heterogeneity in adult cardiac myocytes. J. Mol. Cell Cardiol. 136, 72–84. 10.1016/j.yjmcc.2019.08.013 31491377 PMC7173146

[B94] Lv Y. Cheng L. Peng F. (2022). Compositions and functions of mitochondria-associated endoplasmic reticulum membranes and their contribution to cardioprotection by exercise preconditioning. Front. Physiol. 13, 910452. 10.3389/fphys.2022.910452 35733995 PMC9207531

[B95] Ma Q. (2013). Role of nrf2 in oxidative stress and toxicity. Annu. Rev. Pharmacol. Toxicol. 53, 401–426. 10.1146/annurev-pharmtox-011112-140320 23294312 PMC4680839

[B96] Ma Y. Kuang Y. Bo W. Liang Q. Zhu W. Cai M. (2021). Exercise training alleviates cardiac fibrosis through increasing fibroblast growth factor 21 and regulating TGF-β1-Smad2/3-MMP2/9 signaling in mice with myocardial infarction. Int. J. Mol. Sci. 22, 12341. 10.3390/ijms222212341 34830222 PMC8623999

[B97] Ma X. Gao H. Wang Z. Zhu D. Dai W. Wu M. (2025a). Beneficial effects of different types of exercise on diabetic cardiomyopathy. Biomolecules 15, 1223. 10.3390/biom15091223 41008530 PMC12466945

[B98] Ma Z. Cen Y. Xun W. Mou C. Yu J. Hu Y. (2025b). Exercise enhances cardiomyocyte mitochondrial homeostasis to alleviate left ventricular dysfunction in pressure overload induced remodelling. Sci. Rep. 15, 11698. 10.1038/s41598-025-95637-z 40188200 PMC11972341

[B99] Marcil M. Bourduas K. Ascah A. Burelle Y. (2006). Exercise training induces respiratory substrate-specific decrease in Ca2+-induced permeability transition pore opening in heart mitochondria. Am. J. Physiol. Heart Circ. Physiol. 290, H1549–H1557. 10.1152/ajpheart.00913.2005 16284229

[B100] Memme J. M. Hood D. A. (2020). Molecular basis for the therapeutic effects of exercise on mitochondrial defects. Front. Physiol. 11, 615038. 10.3389/fphys.2020.615038 33584337 PMC7874077

[B101] Meng M. (2017). Digitoflavone (DG) attenuates LPS-induced acute lung injury through reducing oxidative stress and inflammatory response dependent on the suppression of TXNIP/NLRP3 and NF-κB. Biomed. Pharmacother. 94, 712–725. 10.1016/j.biopha.2017.07.001 28800542

[B102] Mozdy A. D. Mccaffery J. M. Shaw J. M. (2000). Dnm1p GTPase-mediated mitochondrial fission is a multi-step process requiring the novel integral membrane component Fis1p. J. Cell Biol. 151, 367–380. 10.1083/jcb.151.2.367 11038183 PMC2192649

[B103] Nguyen P. Le L. K. Ananthapavan J. Gao L. Dunstan D. W. Moodie M. (2022). Economics of sedentary behaviour: a systematic review of cost of illness, cost-effectiveness, and return on investment studies. Prev. Med. 156, 106964. 10.1016/j.ypmed.2022.106964 35085596

[B104] No M. H. Heo J. W. Yoo S. Z. Kim C. J. Park D. H. Kang J. H. (2020). Effects of aging and exercise training on mitochondrial function and apoptosis in the rat heart. Pflugers Arch. 472, 179–193. 10.1007/s00424-020-02357-6 32048000

[B105] Okoye C. N. Koren S. A. Wojtovich A. P. (2023). Mitochondrial complex I ROS production and redox signaling in hypoxia. Redox Biol. 67, 102926. 10.1016/j.redox.2023.102926 37871533 PMC10598411

[B106] Otten A. B. C. Kamps R. Lindsey P. Gerards M. Pendeville-Samain H. Muller M. (2020). Tfam knockdown results in reduction of mtDNA copy number, OXPHOS deficiency and abnormalities in zebrafish embryos. Front. Cell Dev. Biol. 8, 381. 10.3389/fcell.2020.00381 32596237 PMC7303330

[B107] Park J. Y. Wang P. Y. Matsumoto T. Sung H. J. Ma W. Choi J. W. (2009). p53 improves aerobic exercise capacity and augments skeletal muscle mitochondrial DNA content. Circ. Res. 105, 705–712. 10.1161/circresaha.109.205310 19696408 PMC2761626

[B108] PasmiñO G. Paredes M. Silva H. (2024). Effects of high-intensity swimming interval training on area, perimeter, circularity index and phenotype of cardiac mitochondrial ultrastructure in sprague dawley rats. Life (Basel) 14, 984. 10.3390/life14080984 39202726 PMC11355701

[B109] Peoples J. N. Saraf A. Ghazal N. Pham T. T. Kwong J. Q. (2019). Mitochondrial dysfunction and oxidative stress in heart disease. Exp. Mol. Med. 51, 1–13. 10.1038/s12276-019-0355-7 31857574 PMC6923355

[B110] Perera N. Deo M. Tegegne S. Tham Y. K. Mellett N. A. Velagic A. (2025). Influence of diet-induced obesity and voluntary exercise training on cardiac lipids and mitochondrial function in mice. J. Sport Health Sci. 15, 101095. 10.1016/j.jshs.2025.101095 41101703 PMC12811436

[B111] Popoiu T. A. Maack C. Bertero E. (2023). Mitochondrial calcium signaling and redox homeostasis in cardiac health and disease. Front. Mol. Med. 3, 1235188. 10.3389/fmmed.2023.1235188 39086688 PMC11285591

[B112] Protasoni M. Zeviani M. (2021). Mitochondrial structure and bioenergetics in normal and disease conditions. Int. J. Mol. Sci. 22, 586. 10.3390/ijms22020586 33435522 PMC7827222

[B113] Quindry J. C. Michalak R. E. (2025). Exercise-induced cardioprotection: from endogenous to exogenous mechanisms. Sports Med. Health Sci. 7, 366–374. 10.1016/j.smhs.2025.03.009 40936660 PMC12421174

[B114] Riehle C. Wende A. R. Zhu Y. Oliveira K. J. Pereira R. O. Jaishy B. P. (2014). Insulin receptor substrates are essential for the bioenergetic and hypertrophic response of the heart to exercise training. Mol. Cell Biol. 34, 3450–3460. 10.1128/mcb.00426-14 25002528 PMC4135616

[B115] Rodrigues E. A. Lima A. R. R. Gomes M. J. Souza L. M. Pontes T. H. D. Pagan L. U. (2023). Influence of isolated resistance exercise on cardiac remodeling, myocardial oxidative stress, and metabolism in infarcted rats. Antioxidants (Basel) 12, 896. 10.3390/antiox12040896 37107271 PMC10135620

[B116] Rurik J. G. Aghajanian H. Epstein J. A. (2021). Immune cells and immunotherapy for cardiac injury and repair. Circ. Res. 128, 1766–1779. 10.1161/circresaha.121.318005 34043424 PMC8171813

[B117] Sabouri M. Hatami E. Pournemati P. Shabkhiz F. (2021). Inflammatory, antioxidant and glycemic status to different mode of high-intensity training in type 2 diabetes mellitus. Mol. Biol. Rep. 48, 5291–5304. 10.1007/s11033-021-06539-y 34228273

[B118] Sanford J. A. Nogiec C. D. Lindholm M. E. Adkins J. N. Amar D. Dasari S. (2020). Molecular transducers of physical activity consortium (MoTrPAC): mapping the dynamic responses to exercise. Cell 181, 1464–1474. 10.1016/j.cell.2020.06.004 32589957 PMC8800485

[B119] Santos-Alves E. Marques-Aleixo I. Rizo-Roca D. Torrella J. R. Oliveira P. J. MagalhãES J. (2015). Exercise modulates liver cellular and mitochondrial proteins related to quality control signaling. Life Sci. 135, 124–130. 10.1016/j.lfs.2015.06.007 26135624

[B120] Saris N. E. Sirota T. V. Virtanen I. Niva K. Penttilä T. Dolgachova L. P. (1993). Inhibition of the mitochondrial calcium uniporter by antibodies against a 40-kDa glycoproteinT. J. Bioenerg. Biomembr. 25, 307–312. 10.1007/bf00762591 7688718

[B121] Scarpulla R. C. (2011). Metabolic control of mitochondrial biogenesis through the PGC-1 family regulatory network. Biochim. Biophys. Acta 1813, 1269–1278. 10.1016/j.bbamcr.2010.09.019 20933024 PMC3035754

[B122] Schulz R. SchlüTER K. D. (2023). Importance of mitochondria in cardiac pathologies: focus on uncoupling proteins and monoamine oxidases. Int. J. Mol. Sci. 24, 6459. 10.3390/ijms24076459 37047436 PMC10095304

[B123] Shi Y. Zhang H. Huang S. Yin L. Wang F. Luo P. (2022). Epigenetic regulation in cardiovascular disease: mechanisms and advances in clinical trials. Signal Transduct. Target Ther. 7, 200. 10.1038/s41392-022-01055-2 35752619 PMC9233709

[B124] Shin C. S. Meng S. Garbis S. D. Moradian A. Taylor R. W. Sweredoski M. J. (2021). LONP1 and mtHSP70 cooperate to promote mitochondrial protein folding. Nat. Commun. 12, 265. 10.1038/s41467-020-20597-z 33431889 PMC7801493

[B125] Silver J. L. Lamon S. Loke S. Mazzarino G. Croft L. Ziemann M. (2025). Skeletal muscle mitochondria contain nuclear-encoded RNA species prior to and following adaptation to exercise training in rats. Faseb J. 39, e70702. 10.1096/fj.202500157R 40577041 PMC12204305

[B126] Starnes J. W. Taylor R. P. (2007). Exercise-induced cardioprotection: endogenous mechanisms. Med. Sci. Sports Exerc 39, 1537–1543. 10.1249/mss.0b013e3180d099d4 17805086

[B127] Starnes J. W. Barnes B. D. Olsen M. E. (2007). Exercise training decreases rat heart mitochondria free radical generation but does not prevent Ca2+-induced dysfunction. J. Appl. Physiol. (1985) 102, 1793–1798. 10.1152/japplphysiol.00849.2006 17303708

[B128] Sun Q. Karwi Q. G. Wong N. Lopaschuk G. D. (2024). Advances in myocardial energy metabolism: metabolic remodelling in heart failure and beyond. Cardiovasc Res. 120, 1996–2016. 10.1093/cvr/cvae231 39453987 PMC11646102

[B129] Suvorava T. Cortese-Krott M. M. (2018). Exercise-induced cardioprotection *via* eNOS: a putative role of red blood cell signaling. Curr. Med. Chem. 25, 4457–4474. 10.2174/0929867325666180307112557 29521199

[B130] Takeuchi A. Matsuoka S. (2021). Physiological and pathophysiological roles of mitochondrial Na(+)-Ca(2+) exchanger, NCLX, in hearts. Biomolecules 11, 1876. 10.3390/biom11121876 34944520 PMC8699148

[B131] Tocantins C. Martins J. D. Rodrigues Ó M. Grilo L. F. Diniz M. S. Stevanovic-Silva J. (2023). Metabolic mitochondrial alterations prevail in the female rat heart 8 weeks after exercise cessation. Eur. J. Clin. Invest 53, e14069. 10.1111/eci.14069 37525474

[B132] Tokuyama T. Yanagi S. (2023). Role of mitochondrial dynamics in heart diseases. Genes (Basel) 14, 1876. 10.3390/genes14101876 37895224 PMC10606177

[B133] Toldo S. Abbate A. (2024). The role of the NLRP3 inflammasome and pyroptosis in cardiovascular diseases. Nat. Rev. Cardiol. 21, 219–237. 10.1038/s41569-023-00946-3 37923829 PMC11550901

[B134] Tuon T. Souza P. S. Santos M. F. Pereira F. T. Pedroso G. S. Luciano T. F. (2015). Physical training regulates mitochondrial parameters and neuroinflammatory mechanisms in an experimental model of parkinson's disease. Oxid. Med. Cell Longev. 2015, 261809. 10.1155/2015/261809 26448816 PMC4581546

[B135] Vercellino I. Sazanov L. A. (2022). The assembly, regulation and function of the mitochondrial respiratory chain. Nat. Rev. Mol. Cell Biol. 23, 141–161. 10.1038/s41580-021-00415-0 34621061

[B136] Viloria M. A. D. Li Q. Lu W. Nhu N. T. Liu Y. Cui Z. Y. (2022). Effect of exercise training on cardiac mitochondrial respiration, biogenesis, dynamics, and mitophagy in ischemic heart disease. Front. Cardiovasc Med. 9, 949744. 10.3389/fcvm.2022.949744 36304547 PMC9592995

[B137] Wan W. Zhang L. Lin Y. Rao X. Wang X. Hua F. (2023). Mitochondria-derived peptide MOTS-c: effects and mechanisms related to stress, metabolism and aging. J. Transl. Med. 21, 36. 10.1186/s12967-023-03885-2 36670507 PMC9854231

[B138] Wang J. Liu S. Meng X. Zhao X. Wang T. Lei Z. (2024). Exercise inhibits doxorubicin-induced cardiotoxicity *via* regulating B cells. Circ. Res. 134, 550–568. 10.1161/circresaha.123.323346 38323433 PMC11233173

[B139] Waseem R. Shamsi A. Mohammad T. Hassan M. I. Kazim S. N. Chaudhary A. A. (2022). FNDC5/Irisin: Physiology and pathophysiology. Molecules 27, 1118. 10.3390/molecules27031118 35164383 PMC8838669

[B140] Wei Z. Ahmad M. Chen R. Fatima S. Shah S. (2025). High-intensity interval training improves mitochondrial function and attenuates cardiomyocytes damage in ischemia-reperfusion. Int. J. Cardiol. Heart Vasc. 60, 101756. 10.1016/j.ijcha.2025.101756 40756748 PMC12314338

[B141] Xi Y. Hao M. Liang Q. Li Y. Gong D. W. Tian Z. (2021). Dynamic resistance exercise increases skeletal muscle-derived FSTL1 inducing cardiac angiogenesis *via* DIP2A-Smad2/3 in rats following myocardial infarction. J. Sport Health Sci. 10, 594–603. 10.1016/j.jshs.2020.11.010 33246164 PMC8500809

[B142] Xiong Y. Xu J. Cao W. Zhang J. Feng Z. Cao K. (2022). Hydroxytyrosol improves strenuous exercise-associated cardiac pathological changes *via* modulation of mitochondrial homeostasis. Food Funct. 13, 8676–8684. 10.1039/d2fo00839d 35904366

[B143] Xu H. X. Cui S. M. Zhang Y. M. Ren J. (2020). Mitochondrial Ca(2+) regulation in the etiology of heart failure: physiological and pathophysiological implications. Acta Pharmacol. Sin. 41, 1301–1309. 10.1038/s41401-020-0476-5 32694759 PMC7608470

[B144] Yamada T. Kimura I. Ashida Y. Tamai K. Fusagawa H. Tohse N. (2021). Larger improvements in fatigue resistance and mitochondrial function with high-than with low-intensity contractions during interval training of mouse skeletal muscle. Faseb J. 35, e21988. 10.1096/fj.202101204R 34665879

[B145] Yan Z. Lira V. A. Greene N. P. (2012). Exercise training-induced regulation of mitochondrial quality. Exerc Sport Sci. Rev. 40, 159–164. 10.1097/JES.0b013e3182575599 22732425 PMC3384482

[B146] Yang Z. Wang L. Yang C. Pu S. Guo Z. Wu Q. (2021). Mitochondrial membrane remodeling. Front. Bioeng. Biotechnol. 9, 786806. 10.3389/fbioe.2021.786806 35059386 PMC8763711

[B147] Yang M. Xiao Z. Chen Z. Ru Y. Wang J. Jiang J. (2022). S100A1 is involved in myocardial injury induced by exhaustive exercise. Int. J. Sports Med. 43, 444–454. 10.1055/a-1642-8352 34688220

[B148] Yao D. Li Y. Zeng S. Li Z. Shah Z. Song B. (2022). Short-form OPA1 is a molecular chaperone in mitochondrial intermembrane space. Sci. China Life Sci. 65, 227–235. 10.1007/s11427-021-1962-0 34480695

[B149] Yoo S. Z. No M. H. Heo J. W. Park D. H. Kang J. H. Kim J. H. (2019). Effects of acute exercise on mitochondrial function, dynamics, and mitophagy in rat cardiac and skeletal muscles. Int. Neurourol. J. 23, S22–S31. 10.5213/inj.1938038.019 30832464 PMC6433208

[B150] Yuan S. Kuai Z. Zhao F. Xu D. Wu W. (2025a). Improving effect of physical exercise on heart failure: reducing oxidative stress-induced inflammation by restoring Ca(2+) homeostasis. Mol. Cell Biochem. 480, 2471–2486. 10.1007/s11010-024-05124-8 39365389

[B151] Yuan S. Ye Q. Qin R. (2025b). Cardioepigenetics in action: aerobic exercise-induced modulation of miRNAs, lncRNAs, and chromatin remodeling in cardiovascular disease. Front. Cardiovasc Med. 12, 1579352. 10.3389/fcvm.2025.1579352 40822014 PMC12354388

[B152] Zanini G. Selleri V. Malerba M. Solodka K. Sinigaglia G. Nasi M. (2023). The role of Lonp1 on mitochondrial functions during cardiovascular and muscular diseases. Antioxidants (Basel) 12, 598. 10.3390/antiox12030598 36978846 PMC10045650

[B153] Zhang D. Wang F. Li P. Gao Y. (2022). Mitochondrial Ca(2+) homeostasis: emerging roles and clinical significance in cardiac remodeling. Int. J. Mol. Sci. 23, 3025. 10.3390/ijms23063025 35328444 PMC8954803

[B154] Zhang M. Alemasi A. Zhao M. Xu W. Zhang Y. Gao W. (2023). Exercise training attenuates acute β-Adrenergic receptor activation-induced cardiac inflammation *via* the activation of AMP-activated protein kinase. Int. J. Mol. Sci. 24, 9263. 10.3390/ijms24119263 37298222 PMC10252890

[B155] Zhao H. X. Zhang Z. Zhou H. L. Hu F. Yu Y. (2020). Exercise training suppresses Mst1 activation and attenuates myocardial dysfunction in mice with type 1 diabetes. Can. J. Physiol. Pharmacol. 98, 777–784. 10.1139/cjpp-2020-0205 32687725

[B156] Zhuo C. Xin J. Huang W. Zhang D. Yan X. Li R. (2023). Irisin protects against doxorubicin-induced cardiotoxicity by improving AMPK-Nrf2 dependent mitochondrial fusion and strengthening endogenous anti-oxidant defense mechanisms. Toxicology 494, 153597. 10.1016/j.tox.2023.153597 37499777

[B157] Zong Y. Li H. Liao P. Chen L. Pan Y. Zheng Y. (2024). Mitochondrial dysfunction: mechanisms and advances in therapy. Signal Transduct. Target Ther. 9, 124. 10.1038/s41392-024-01839-8 38744846 PMC11094169

[B158] Zou R. Tao J. Qiu J. Shi W. Zou M. Chen W. (2021). Ndufs1 deficiency aggravates the mitochondrial membrane potential dysfunction in pressure overload-induced myocardial hypertrophy. Oxid. Med. Cell Longev. 2021, 5545261. 10.1155/2021/5545261 33763166 PMC7952157

